# Deep Learning Assisted Proton Pure Shift NMR Spectroscopy

**DOI:** 10.1021/jacs.5c22860

**Published:** 2026-03-02

**Authors:** Veera Mohana Rao Kakita, D. Flemming Hansen

**Affiliations:** † The Francis Crick Institute, 1 Midland Road, London NW1 1AT, U.K.; ‡ Department of Structural and Molecular Biology, Division of Biosciences, 4919 University College London, London WC1E 6BT, U.K.

## Abstract

Nuclear magnetic resonance spectroscopy (NMR) plays a key role for the analysis of a plethora of molecules, including natural products and drug-like organic molecules. For such cases, ^1^H NMR spectra have proven imperative because of their high sensitivity and atomic resolution. However, these spectra are complicated by overlapped complex multiplet patterns. Here we show a deep-learning approach, which transforms spin–echo modulated ^1^H NMR spectra into highly sensitive and high-resolution singlet NMR spectra, that is, virtual homonuclear decoupled pure shift spectra. The approach was evaluated on experimental NMR spectra of complex organic compounds, where it outperforms current methods. The method also predicts uncertainties of the transformation and therefore allows for quantifications, which is a key strength of NMR. We believe that our approach will provide significant advantages when characterizing low-sensitivity samples and systems with exchangeable protons, where signals are not observed in traditional pure-shift spectra and substantial overlaps hamper analysis from conventional spectra.

## Graphical abstract

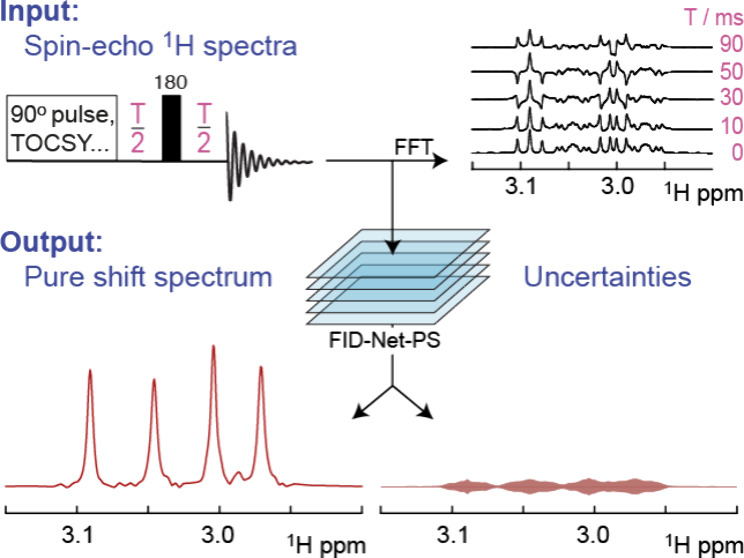

Proton nuclear magnetic resonance (^1^H NMR) spectroscopy provides critical structural insights on organic molecules.^1,2^ However, the resolution of ^1^H NMR spectra suffers significantly from the presence of overlapping homonuclear scalar coupling multiplets. These can, in favorable cases, be transformed into resolved singlets (pure shifts) using homonuclear decoupling NMR methods.^3–18^ This improvement in the spectral resolution can be up to an order of magnitude, however, at a trade-off of spectral sensitivity of up to 2 orders of magnitude if a homonuclear broadband decoupling scheme is considered.^10,14,15^ Although homonuclear band-selective decoupling can offer full sensitivity, it is not applicable if any scalarly coupled spins coexist within the chosen frequency range.^7,17,18^ For complex organic molecules it is, therefore, necessary to consider broadband decoupling methods to obtain the required pure shift spectrum experimentally. Conversely, it is not possible to detect broad or low-sensitive signals using these broadband decoupling schemes. Similarly, long-pulse based pure shift NMR fails to detect exchangeable protons. This, in turn, calls for new strategies to improve the spectral sensitivity of pure shift NMR experiments.

Recently there have been attempts to improve the sensitivity of pure shift experiments, including nonuniform sampling (NUS), with either conventional or deep learning processing,^19–23^ as well as Hadamard transformations.^24^ Incorporating these strategies partially recovers the signal sensitivities per unit of experimental time. Hyperpolarisation,^25^ multislice excitation,^26,27^ and frequency shifting^28^ have also been explored to recover the signal sensitivity of pure shift NMR.

Having a method or strategy that provides high-sensitivity pure shift NMR spectra would solve a wide-range of challenges regarding the detection of valuable spectral information, including quantifications of mixtures. In the current era of artificial intelligence and deep learning it is natural to leverage these tools to improve the sensitivity of pure shift NMR and NMR in general.^29^ It is worth noting that some recent deep-learning applications in NMR span automation of protein structural analysis^30,31^ and improvement of spectral resolution in solid-state NMR.^32^ A few deep learning methods have been demonstrated to reconstruct NUS spectra.^33–36^ Whereas the model JRESRGAN^37^ was developed for peak deconvolution, the FID-Net deep learning architecture makes advances possible in NMR spectral processing of proteins.^38–41^ Recently, it was demonstrated for organic molecules and using spin–echo spectra that the model SE2CSNet^42^ can predict ^1^H NMR chemical shifts in the form of a list of values or as a ‘pseudospectrum’ consisting of vertical lines all with unit intensities. Since this strategy does not generate peaks with realistic line widths and intensities it unfortunately unsuitable for quantitative analyses.

Below we present a combined NMR and deep learning approach that provides high-sensitivity, high-resolution, pure-shift ^1^H spectra of small molecules along with point-to-point uncertainties. The presented strategy employs a new neural network architecture with ideas taken from the previous FID-Net-2.^40^ The new architecture is named FID-Net-PS and in comparison to the FID-Net-2 architecture, the FID-Net-PS input has been tailored and consists of five 1D-spectra that are analyzed simultaneously (Figure 1), where the scalar couplings have been evolved for different times. Whereas FID-Net-2 ^40^ was designed for decoupling of simple doublet and triplet patterns, the FID-Net-PS architecture can decouple any type of complex multiplets. The full model is described in Figure S1.

**Figure 1:**
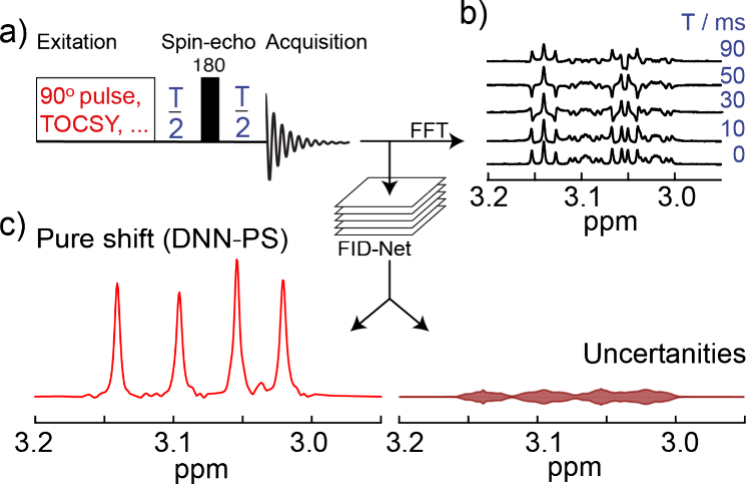
A schematic workflow for obtaining pure shift, i.e. virtually homonuclear decoupled spectra, using the deep neural network FID-Net-PS. a) Representation of the spin–echo pulse sequence and implementation with 1D and 2D NMR. b) Representative spin–echo modulated frequency-domain spectra, recorded with five different spin–echo delays, T, and shown over an expanded chemical shift region. c) The spin–echo spectra serve as input to the FID-Net-PS model, which produces the pure shift (DNN-PS) spectrum and associated uncertainties.

Figure 1 depicts a schematic representation of the process adopted here for generating virtually decoupled pure-shift spectra. The spin–echo-based pulse sequences, Figure 1a, generate *J*-modulated multiplet patterns in 1*D*/2D ^1^H NMR spectra, in the direct dimension. Thus, each experiment consists of recording five spectra at spin–echo evolution durations of 0, 10, 30, 50, and 90 ms. These points can be seen as the sparsely sampled points in a J-res experiment with an exponential weighing, Figure 1b, which create a unique pattern in the data for each multiplet type. These unique patterns are used as input to the FID-Net-PS model and allow the DNN to separate the different multiplet types and virtually decouple these (Figure 1c). The FID-Net-PS model, Figure 1 and Figure S1, was trained fully on synthetic data, as was also done for previous FID-Net models^38–41^ (see Supporting Information). The target for the training is the corresponding synthetic pure shift NMR spectra, that is, the spectrum calculated with all scalar couplings set to zero.

Initially, the FID-Net-PS model was evaluated using synthetic data, Figure S2. The model was trained across a wide range of noise levels of the input spectra, however, initially it tended to underpredict the uncertainties. This behavior is likely due to partial signal masking at higher noise levels and conservative predictions in low-noise regions, which arise from the model averaging over the full distribution of noise during training. To enable its application across a wide range of noise levels, a calibration curve was generated, Figure S3. The predicted uncertainties derived through this calibration are valid for signal-to-noise levels of 20:1 to 10^5^:1, which span most experimental cases.

The trained FID-Net-PS model was subsequently evaluated on experimental NMR spectra of kanamycin (34.3 mM and 50.0 μM), estradiol, a mixture of kanamycin and glucose, on a mixture of 11 amino acids, and trichostatin-A (TSA). Figure 2 compares the conventional 1D ^1^H NMR spectrum, the PSYCHE,^14^ and FID-Net-PS generated DNN-PS spectra obtained on the kanamycin sample (34.3 mM). From Figure 2c, it is seen that the PSYCHE approach resolves the overlap at the expense of spectral sensitivity, whereas the DNN-PS spectrum has considerably improved spectral resolution without compromising sensitivity.

**Figure 2:**
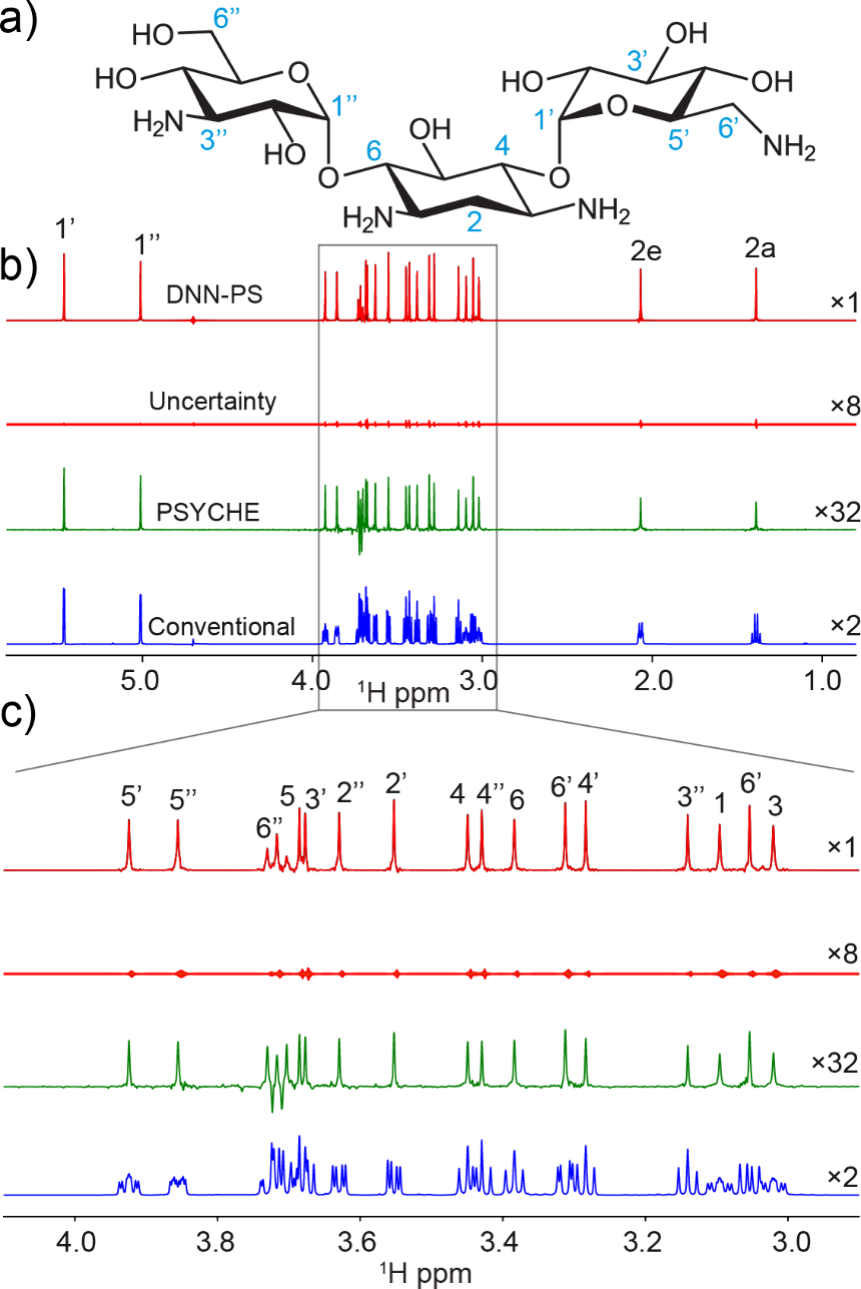
a) Chemical structure of kanamycin. b) Comparison of a conventional 1D ^1^H NMR spectrum (blue), a PSYCHE (green), and an FID-Net-PS DNN-PS processed spectrum along with uncertainties (red), obtained from a 34.3 mM sample of kanamycin in D_2_O and recorded at 800 MHz. c) Zoom of region with highly overlapped peaks. As with other conventional pure shift methods, FID-Net-PS still suffers from limitations with strongly coupled spin networks (see 6′′ near 3.7 ppm), since the training data were generated only based on the approximation of semiweakly coupled spin systems (see text).

For the strongly coupled AB spin system in Figure 2c (6′′ near 3.7 ppm), the PSYCHE approach generates significantly distorted patterns, however, these are substantially reduced in the DNN-PS spectrum. It is also clear that the FID-Net-PS model effectively works by reconstructing the projection of a sparsely sampled J-res, which leads to the two shoulder peaks. The evaluation of the FID-Net-PS approach was also carried out on estradiol, Figure S4. Estradiol, like kanamycin, has a relatively low spectral resolution in the conventional 1D ^1^H NMR spectrum, which becomes well-resolved in the DNN-PS spectrum. From these two examples it is evident that the FID-Net-PS model outperforms both conventional and PSYCHE NMR methods, in terms of spectral sensitivity and resolution. The predicted uncertainties are useful for evaluating ambiguities in the predictions for all resonances and these are, as expected, higher for noisy and overlapped spectral regions. A sudden spike of uncertainties would indicate that the predicted resonance may be an artifact, however, none of these are seen in our results.

Judging the sensitivity of spectra obtained from nonlinear transformations, such as DNNs, need special considerations because the ‘noise’ is not uniform and predicted uncertainties thus need to be used as proxy for the noise. An attempt was made to evaluate the sensitivity of kanamycin spectra recorded using PSYCHE or DNN-PS methods with addition of white noise (see Supporting Information, Figures S5, and S6). During the noise addition process, most of the peaks were retained in the DNN-PS spectra, however, several resonances disappeared in the PSYCHE spectra. This demonstrates that the sensitivity of FID-Net-PS is significantly higher than that of PSYCHE and that the sensitivity aligns with the predicted uncertainties.

One-dimensional ^1^H NMR spectra are often used for mixture quantitation, and it is therefore of interest to evaluate how quantitative the transformation performed by the trained FID-Net-PS model is. The conventional 1D ^1^H spectra of kanamycin and glucose mixture are shown in Figure 3a. The spectral resolution of this sample is at a level where general quantifications are very challenging, however, all resonances are well-resolved in the DNN-PS spectrum (see Figure S7 for chemical shift assignments). Titration of additional kanamycin enables for an assessment of the quantitative nature of FID-Net-PS. For the initial quantification, only well-separated resonances were considered, Figure S8, to allow for a clear comparison of quantifications from different experiments. Subsequently, the evaluation was extended to highly overlapped peaks, Figure 3a. The measured signal integrals for kanamycin and glucose were monitored against the corresponding constituent concentration, Figure 3b. They are found to be well in agreement for the DNN-PS spectrum which is evident from the higher Pearson correlation coefficient (*r*). Furthermore, the sensitivity of the spectra can be judged from the normalized RMSD (*ρ̃*). Overall, the DNN-PS spectra can be utilized to quantify chemical constituents at ultrahigh resolution and high sensitivity, with predicted uncertainties used as error bars.

**Figure 3:**
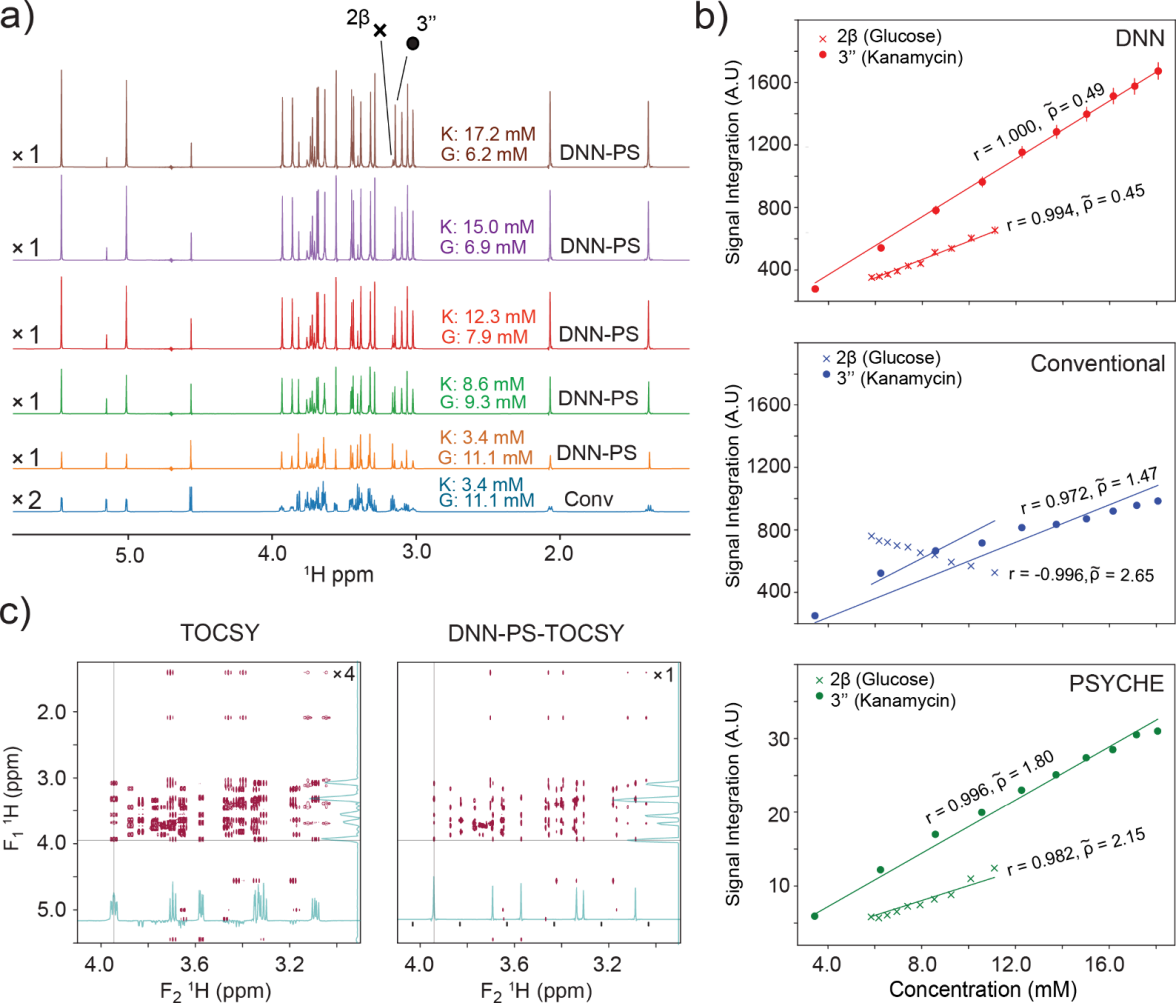
a) The conventional ^1^H NMR and DNN-PS spectra of kanamycin (K) and glucose (G) mixtures recorded at different concentration ratios. A substantial resolution improvement is seen in the DNN-PS spectra compared to the traditional spectrum. b) The peaks obtained from the conventional (blue), DNN-PS (red and the error bars are the predicted uncertainties), and PSYCHE (green), are integrated and presented against concentrations (kanamycin (filled circles) and glucose (cross)). Pearson correlation coefficients, r, for PSYCHE and DNN-PS are high. On the other hand, for glucose resonances from the conventional spectrum, the measured correlation coefficients r is found to be negative due to the severe signal overlap. Therefore, in this case, the conventional experiments fail. Another measure of the performance is the normalized RMSD, $\mathrm{\rho ̃}=\sqrt{t_{\mathrm{acq}}/t_{\mathrm{acq},\mathrm{conv}}}$ RMSD/slope, which normalizes the RMSD both with respect to raw intensities and with respect to the time required for the experiment, *t*_acq_, relative to the conventional 1D spectrum, *t*_acq,conv_. (c) The applicability of the DNN-PS approach is demonstrated along the direct data acquisition dimensions of a 2D -^1^H–^1^H TOCSY acquired on this mixture. The conventional 2D-TOCSY has significant overlaps, however, all peaks are resolved in the DNN-PS spectrum. The residual negative intensities observed in the TOCSY projections (for 5′) arise from baseline imperfections; these artifacts are completely removed in the DNN-PS projections.

Figure 3c shows the applications of the FID-Net-PS model for processing 2D-TOCSY. Before submitting spin–echo modulated TOCSY to the FID-Net-PS model, Fourier transformation was performed along the indirect dimension and subsequently all these slices were processed independently with the FID-Net-PS model akin to 1d-processing. The resultant two-dimensional DNN-PS TOCSY facilitates unambiguous correlations when compared with the conventional TOCSY recorded on the mixture of kanamycin and glucose (Figure 3c). Figures S9–S15 show some additional FID-Net-PS applications on complex molecules, mixtures, and systems consisting of conformationally and chemically exchanging protons. These results clearly demonstrate that the FID-Net-PS method outperforms conventional methods in obtaining pure shift spectra with high sensitivity in 1D, 2D-TOCSY, and (selective) 1D-TOCSY experiments.

The presented FID-Net-PS method is an alternative approach to the conventional pure shift NMR for obtaining virtually decoupled spectra, both in 1D and in 2D and at high spectral sensitivity. The FID-Net-PS model was trained on synthetic spin–echo spectra, where the modulated scalar couplings aid the model in learning to transform highly complex multiplet patterns into singlet peaks. The applications of this FID-Net-PS model have been demonstrated on complex organic molecules such as kanamycin, estradiol, a mixture of 11 amino acids, a very low concentrated kanamycin, and a mixture with exchangeable protons. Since the FID-Net-PS model not only performs virtual decoupling but also predicts uncertainties of the transformation, using these as error bars makes quantifications reliable as shown with analyses of the kanamycin and glucose mixtures. A current limitation of the FID-Net-PS model is its suboptimal handling of strongly coupled peaks, e.g. 6′′ in Figure 2c, and these are situations that we are currently working on improving. Overall, the benefits associated with the presented method will not be limited to 1D ^1^H NMR spectra, but as we have shown can easily be extended to higher dimensions. It is anticipated that the main strength of the FID-Net-PS approach is when analyzing low-sensitivity samples, where strong overlaps are observed in conventional spectra and no signals are observed in traditional pure-shift spectra.

## Supplementary Material



## Data Availability

Scripts for virtual decoupling of 1D ^1^H spin–echo spectra using FID-Net-PS, including pretrained networks and examples, are available to download from https://github.com/hansenlab-ucl/DNN_PS. Scripts are available both (*i*) for processing within Bruker TopSpin on the NMR spectrometer and (*ii*) for processing off the spectrometer.

## Notes

Complete materials and methods, as well as 1D, selective 1D-TOCSY, and 2D-TOCSY NMR spectra obtained using conventional pure shift and FID-Net-PS processing. (PDF)

## Abbreviations

NMR: Nuclear Magnetic Resonance
DNN: Deep Neural Network
