# Supplementary material for: Deep Learning Assisted Proton Pure Shift NMR Spectroscopy
Source: J Am Chem Soc. 2026 Mar 2;148(9):9226–30. doi: 10.1021/jacs.5c22860 (PMC12983296; doi:10.1021/jacs.5c22860)
Supplement: Supplementary file 1 [file ja5c22860_si_001.pdf]

Supporting Information

Deep Learning Assisted Proton Pure Shift NMR Spectroscopy

Veera Mohana Rao Kakita[a,b,*] and D. Flemming Hansen[a,b,*]

[a] The Francis Crick Institute, 1 Midland Road, London, NW1 1AT, UK.
[b] Department of Structural and Molecular Biology, Division of Biosciences, University College London, London, WC1E 6BT, UK.

*Correspondence should be addressed to D.F.H. (flemming.hansen@crick.ac.uk or d.hansen@ucl.ac.uk) or V.M.R.K. (v.kakita@ucl.ac.uk)

Supporting Materials and Methods

1. Synthetic ^{1}H NMR spectra for the FID-Net-PS model training:

The mathematical formula shown below was employed in conjunction with the parameters from Table. S1 to synthesise the spin-echo NMR spectra as well as the target pure-shift spectra. It is noted that this model includes non-weak coupled spins by way of introducing roofing (*k*).

$$S(t+T) = \left(\sum_{n=1}^{m} S_0 e^{i(\omega_n+\phi_n)t} e^{-R_2 t} \prod \left(\cos(\pi J_n(t+T)) + i(1-k)\sin(\pi J_n(t+T))\right) e^{-R_{2-inhom}t} + S_{noise}\right.$$

Where,

$S(t+T)$	= spin-echo signal at the echo time of T(s)
S_0	= signal intensity
t	= acquisition time
n	= number of peaks
ω	= chemical shift (rad/sec)
ϕ	= phase of the signal
R_2	= transverse relaxation rate of the signal
J_n	= scalar coupling (Hz)
$R_{2-inhom}$	= relaxation rate due to B_0 inhomogeneity
S_{noise}	= added noise
k	= roofing component in scalar coupling multiplets (0 to 1)

To replicate the ^{1}H-NMR complex spectral patterns (see below), spectra were synthesised with both an overlapped band of signals (condensed band, whose position changes for each set of spectra) and non-overlapped chemical shift resonances (non-condensed band). These spectra served as training data for the pure shift FID-Net-PS DNN model.

$$\text{Number of signals in the condensed band } (cb) = \frac{1}{2}(\text{total number of signals})$$
$$\text{Numbe of signals in the non} - \text{condensed band } (ncb) = \text{total number of signals} - \text{cb}$$
$$\text{Location of the condensed band } (loc) = -\frac{7}{10}SW \text{ to } \frac{7}{10}SW \text{ (uniform distribution)}$$
$$\text{Condensed bandwidth } (con_{ban}) = 0.01 - 0.05 \text{ (uniform distribution)}$$
$$\text{Chemical shifts in cb } (cs_{cb}) = N(loc, con_{ban} \times SW) \text{ (normal distribution)}$$
$$\text{Chemical shifts in ncb } (cs_{ncb}) = N\left(0, \tfrac{1}{4}SW\right) \text{ (normal distribution)}$$
$$\text{Chemical shifts } (cs) = \tanh\left(\frac{2}{SW}(cs_{cb} + cs_{ncb})\right) \times \frac{SW}{2}$$

This final step ensures that chemical shifts do not fall outside the spectral widths.

2. *DNN Pure shift (DNN-PS) FID-Net-PS model training:*

The pure shift FID-Net model (Fig. S1) was developed using Tensorflow 2.12 (python)[1] and trained on 14×10^7 synthetic NMR spectra, with each round having 8×10^3 spectra to update model weights, for a total of 17×10^3 training rounds. Each mini-batch consists of 12 synthetic NMR spectra, and the model was trained on NMRBox virtual machines using three A100 GPUs. For the training, the scheduled Adam optimiser was used with the following parameters

$d_{model} = 20 \times 20 \times 18$, $\beta_1 = 0.9$, $\beta_1 = 0.98$, $\varepsilon = 10^{-9}$, $warmup\ steps = 20000$, $lr_{min} = 2.0 \times 10^{-7}$ and step = counter for batches

The learning rate (lr) was calculated as follows,

$$\text{learning rate (lr)} = \max(lr_{min}, \sqrt{d_{model} \times \frac{1}{100} \times \min\left(\sqrt{step}, step \times \frac{1}{\sqrt{warmup\ steps^3}}\right)}$$

The final layer of FID-Net-PS generates two tensors: the pure shift NMR spectra and their corresponding confidences. To ensure these confidences in the range of 0 to 1, a sigmoidal activation was used, and the predicted uncertainties were computed from the confidence as follows,

$$\sigma = \left(\frac{1}{0.998 \times sigmoid(conf) + 0.001} - 1\right)$$

Training the FID-Net-PS model was done with customised loss functions, similar to that of the loss functions used in the FID-Net-2,[2] where the total loss is the summation of all the losses ($Total\ loss = Loss_1 + Loss_2 + Loss_3$). The $Loss_1$ is the conventional measure-square-error (MSE) loss function, which minimizes the difference between the predicted pure shift NMR spectrum to the target pure shift NMR spectrum, and that eventually helps to predict the pure shift NMR spectrum closer to that of the target spectrum. The sum is calculated over all points in the 1D spectrum, and N is the total number of points.

$$Loss_1 = \frac{1}{N} \sum_i (target_i - predict_i)^2$$

The losses $Loss_2$, and $Loss_3$ are specially designed for the pure shift FID-Net to capture the uncertainties. For $Loss_2$, a value χ_i was initially computed for all the points in the data, wherein, σ_i is the predicted uncertainties. Finally, this χ_i was minimised for capturing the Gaussian distribution of predicted errors. The requirement has been achieved by restraining the 1/2th, 2nd, 3rd, and 7/2th momenta of χ_i as given below,

$$\chi_i = \frac{target_i - predicted_i}{\sigma_i}$$

$$Loss_2 = \sum_{m \in \left\{\frac{1}{2};2;3;\frac{7}{2}\right\}} \left\{\left(\frac{1}{N}\sum_i \chi_i^m\right) - M_m\right\}^2$$

where, $M_{\frac{1}{2}} = \left(-\frac{1}{2}\right)^{\frac{1}{4}} \Gamma\left(\frac{3}{4}\right)/\sqrt{\pi}$, $M_2 = 1$, $M_3 = 0$, $M_{\frac{7}{2}} = 2^{\frac{3}{4}} \Gamma\left(\frac{9}{4}\right)/\sqrt{\pi}$, and $\Gamma()$ is the gamma function.

For calculating the $Loss_3$, the predicted errors were binned into 200 equal parts. For each bin, the average of predicted errors was restrained to the RMSD between the predicted and target data points as shown below.

$$Loss_3 = \frac{1}{N} \sum_{b \in bins} \left\{ \frac{1}{N_b} \sum_{i \in b} \sigma_i - \sqrt{\frac{1}{N_b} \sum_{i \in b} (target_i - predicted_i)^2} \right\}^2$$

Once the pure shift FID-Net-PS model training was completed, this trained model was initially evaluated on synthetic data and then on a set of experimental data recorded on different individual small organic molecules as well as complex mixtures of small molecules. Subsequently the estimation of the uncertainties were calibrated as detailed in Fig S3.

3. FID-Net-PS architecture for predicting pure shift NMR:

Fig. S1 depicts the complete FID-Net-PS architecture used to predict pure shift NMR spectra. The architecture is nearly identical to that of the original FID-Net-PS,[3] however, it includes additional frequency domain refinement steps and can predict uncertainty. As has been described, two additional loss functions are developed to account for uncertainty. This modified FID-Net-PS model is fed spin-echo spectra collected at evolution times of 0 ms, 10 ms, 30 ms, 50 ms, and 90 ms, equivalent to a sparsely sampled *J*-res spectrum, and it will produce two tensors corresponding to the predicted pure shift NMR spectrum and the uncertainties. These spin-echo spectra (J_{HH}-modulated) will enable the model to learn different proton-proton scalar coupling patterns. Without these modulations, the model will be unable to distinguish scalar coupling multiplets originating from different spins of small molecules.

4. Evaluation of FID-Net-PS model on synthetic data:

Fig. S2 depicts the complete evaluation process. Synthetic NMR spectra were used in the first step to evaluate the FID-Net-PS model. Since the synthetic data includes ground-truth pure shift target NMR spectra, this evaluation allows for reliable model assessment. The overall goal of this evaluation is to determine how the trained model behaves across a wide range of noise levels. For this task, 100 sets of spin-echo spectra were generated randomly at each noise level (normalised standard deviation of the noise ranging from 0.0 to 10.0), along with the corresponding target pure shift NMR spectra. The trained FID-Net-PS model takes the spin-echo spectra as input to predict the pure shift NMR spectra, along with the associated uncertainties. The predicted spectra at different noise levels (Fig. S2a, S2b, and S2c, corresponding to noise STD levels of 0.0, 1.0, and 10.0, respectively) are comparable to the target synthetic pure shift spectra.

Plots of RMSD(*predict – target*) *versus* the predicted uncertainties (σ) (for all 100 NMR datasets) indicate that the predicted uncertainties are underestimated, in particularly for large noise levels. This is also evident from the distributions of (*predict – target*)/(*predicted*

uncertainties (σ)), which do not follow normal distributions. In the original FID-Net-2 model demonstrated for aromatic residues, the model was trained on relatively small noise levels. However, in the present case, we aim to make the model robust to a wide range of noise levels. Rather than training multiple FID-Net-PS models at different noise levels, the estimation of uncertainties was calibrated as shown in Fig. S3. This behaviour can be attributed to two complementary effects. At higher noise levels, partial masking of the true signal reduces the model's ability to fully capture the underlying spectral features, leading to underestimated uncertainties. Conversely, for low-noise peaks, the model tends to make conservative predictions, as it has learned to accommodate the broader range of noise encountered during training. Together, these factors reflect the model's effective averaging over the full noise distribution, which, while improving overall robustness, results in systematic underestimation of predicted uncertainties prior to calibration.

5. NMR samples and experiments:

17β-estradiol:
The required sample was prepared by dissolving 20 mg of 17β-estradiol in 1ml of DMSO-D_6, resulting in a concentration of 73.4mM. This sample was utilized to record the requisite 1D-^{1}H, spin-echo, PSYCHE, and TOCSY NMR spectra using an 800 MHz AVANCE-III NMR spectrometer with a cryoprobe.

Experimental details:

1D-^{1}H: The proton 1D spectrum was acquired with a 2s recycle delay and 4 scans. The number of points was 32k, and the spectral width of 8000 Hz resulted in an FID duration of 2.04 s (0.49 Hz spectral resolution). The experiment took around 30s to acquire.

Spin-echo: The spin-echo 1D spectra were acquired with a 2s recycle delay, 4 scans, and five distinct spin-echo evolution times (0ms, 10ms, 30ms, 50ms, and 90ms). The number of points was 32k, and the spectral width of 8000 Hz resulted in an FID duration of 2.04 s (0.49 Hz spectral resolution). The experiment was carried out in pseudo-2D mode and took around 2min and 30s to acquire.

PSYCHE: The PSYCHE pure shift was acquired in pseudo-2D mode with a recycle delay of 2s, 4 scans, and a low-flip angle of 15° for the double frequency swept pulses of 30ms length. The spectral widths in the direct and indirect dimensions were 8000 Hz and 50 Hz, respectively. The direct and indirect dimensions respectively had 16k and 32 number of points. This experiment took around 6min and 30s to acquire. The spectral resolution for the pure shift was 3.1 Hz.

2D-TOCSY: Two-dimensional TOCSY experiments suitable for the FID-Net pure shift processing were acquired in pseudo-3D mode, with a recycle delay of 1s and 4 scans, and dipsi isotropic spinlock mixing set to 60ms. The number of points in the direct and indirect dimensions were 16k and 256, respectively. At a spectral width of 8000 Hz along both dimensions, resulted in spectral resolution of 1 Hz along the direct dimension and 62 Hz along the indirect dimension. Prior to processing the data using FID-Net, Fourier transformation was performed along the indirect dimension, and all indirect slices were processed using the FID-Net model. The experiment took around 3hours and 5min to complete.

Kanamycin:
The requisite sample was prepared by dissolving 20mg of kanamycin in 1ml of D_2O, yielding 34.3 mM concentration. This sample was utilized to record the necessary 1D-^{1}H, spin-echo, PSYCHE, and TOCSY NMR spectra using an 800 MHz AVANCE-III NMR spectrometer equipped with a cryoprobe.

Experimental details:

1D-^{1}H: The proton 1D spectrum was acquired with a 2s recycle delay and 4 scans. The number of points was 32k, with a spectral width of 8100 Hz resulting in an FID duration of 2.02 s (0.49 Hz spectral resolution). The experiment took around 30s to acquire.

Spin-echo: The spin-echo 1D spectra were acquired with a 2s recycle delay, 4 scans, and five distinct spin-echo evolution times (0ms, 10ms, 30ms, 50ms, and 90ms). The number of points was 32k, with a spectral width of 8100 Hz resulting in an FID duration of 2.02 s (0.49 Hz spectral resolution). The experiment was carried out in pseudo-2D mode and took around 2min and 30s to acquire.

PSYCHE: The PSYCHE pure shift was acquired in pseudo-2D mode with a recycle delay of 2s, 4 scans, and a low-flip angle of 15° for the double frequency swept pulses of 30ms length. The spectral widths of the direct and indirect dimensions were 8100 Hz and 50 Hz, respectively. The direct and indirect dimensions respectively had 16k and 32 number of points. This experiment took around 6min and 30s to acquire. The resulting spectral resolution for the pure shift spectrum was 3.1 Hz.

2D-TOCSY: Two-dimensional TOCSY experiments suitable for the FID-Net pure shift processing were acquired in pseudo-3D mode, with a recycle delay of 1s and 4 scans, and dipsi isotropic spinlock mixing set to 60ms. The number of points in the direct and indirect dimensions were 16k and 256, respectively. At a spectral width of 8100 Hz along both dimensions, spectral resolution is 1 Hz along the direct and 63 Hz along the indirect dimensions. Prior to processing the data using FID-Net, Fourier transformation was performed along the indirect dimension, and all indirect slices were processed using the FID-Net model. The experiment took around 3 hours and 13 min to complete.

Kanamycin 50 μM:
A 50 μM Kanamycin sample was prepared from a 34.3 mM stock solution through a series of serial dilutions. This sample was used to acquire the required 1D ^{1}H, spin-echo, and PSYCHE spectra on an 800 MHz Bruker AVANCE III NMR spectrometer equipped with a cryoprobe.

Experimental details:

1D-^{1}H: The proton 1D spectrum was acquired with a 2s recycle delay and 32 scans. The number of points was 32k, with a spectral width of 8100 Hz resulting in an FID duration of 2.02 s (0.49 Hz spectral resolution). The experiment took around 3 min to acquire.

Spin-echo: The spin-echo 1D spectra were acquired with a 2s recycle delay, 32 scans, and five distinct spin-echo evolution times (0ms, 10ms, 30ms, 50ms, and 90ms). The number of points was 32k, with a spectral width of 8100 Hz resulting in an FID duration of 2.02 s (0.49 Hz spectral resolution). The experiment was carried out in pseudo-2D mode and took around 14 min to acquire.

PSYCHE: The PSYCHE pure shift was acquired in pseudo-2D mode with a recycle delay of 2s, 32 scans, and a low-flip angle of 15° for the double frequency swept pulses of 30ms length. The spectral widths of the direct and indirect dimensions were 8100 Hz and 50 Hz, respectively. The direct and indirect dimensions respectively had 8k and 32 number of points. This experiment took around 1 hour 39 min to acquire. The resulting spectral resolution for the pure shift spectrum was 3.1 Hz.

Mixture of kanamycin and glucose:
Kanamycin and glucose stock solutions were prepared separately by dissolving 20 mg/ml in D_2O each. The required volumes were pipetted (50 ul of kanamycin and 50 ul of glucose) and added together, to this, 400 µl of D_2O was also added, yielding a sample concentration of 3.4mM of kanamycin and 11.1mM of glucose, in the mixture sample. On this sample, 1D-^{1}H, spin-echo, PSYCHE, and TOCSY NMR spectra were acquired using an 800 MHz AVANCE-III NMR spectrometer equipped with a cryoprobe.

Keep on adding 50ul of kanamycin stock solution (in each titration) to the above-prepared mixture sample results in kanamycin and glucose concentrations in the following ratios- 6.31mM:10.1mM; 8.6mM:9.3mM; 10.6mM:8.5mM; 12.3mM:7.9mM; 13.7mM:7.4mM; 15.0mM:6.9mM; 16.2mM:9.5mM; 17.2mM:6.2mM; 18.1mM:5.8mM. These samples were utilized for the quantitation using 1D-^{1}H, spin-echo, and PSYCHE spectra recorded on an 800 MHz AVANCE-III NMR spectrometer with a cryoprobe.

Experimental details:

1D-^{1}H: The proton 1D spectrum was acquired with a 2s recycle delay and 4 scans. The number of points was 32k, and the spectral width of 8100 Hz resulted in an FID duration of 2.02s (0.49 Hz of spectral resolution). The experiment took around 30s to acquire.

Spin-echo: The spin-echo 1D spectra were acquired with a 2s recycle delay, 4 scans, and five distinct spin-echo evolution times (0ms, 10ms, 30ms, 50ms, and 90ms). The number of points was 32k, and the spectral width of 8100 Hz resulted in an FID duration of 2.02s (0.49 Hz of spectral resolution). The experiment was carried out in pseudo-2D mode and took around 2min and 30s to acquire.

PSYCHE: The PSYCHE pure shift was acquired in pseudo-2D mode with a recycle delay of 2s, 4 scans, and a low-flip angle of 15° for the double frequency swept pulses of 30ms length. The spectral widths of the direct and indirect dimensions were 8100 and 50 Hz, respectively. The direct and indirect dimensions respectively had 16k and 32 number of points. This experiment took around 6 min and 30s to acquire. The spectral resolution for the pure shift was 3.1 Hz.

2D-TOCSY: Two-dimensional TOCSY experiments suitable for the FID-Net pure shift processing were acquired in pseudo-3D mode, with a recycle delay of 1s and 4 scans, and dipsi isotropic spinlock mixing set to 60 ms. The number of points in the direct and indirect dimensions were 16k and 256, respectively. At a spectral width of 8100 Hz along both dimensions, spectral resolution is 1 Hz along the direct and 63 Hz along the indirect dimensions. Prior to processing the data using FID-Net, Fourier transformation was performed along the indirect dimension, and all indirect slices were processed using the FID-Net model. The experiment took around 3 hours and 13 min to complete.

Selective-1D-TOCSY: The proton selective-1D-TOCSY spectra were acquired with a 2s recycle delay and 4 scans. The number of points was 32k, and the spectral width of 8100 Hz resulted in an FID duration of 2.02s (0.49 Hz of spectral resolution). The experiment took around 30s to complete. Isotropic mixing was produced by using a dipsi spinlock block for 100ms. The requisite spin-state selectivity was obtained during refocusing, with Gaussian shaped pulses utilized for 17.6 ms, corresponding to 50 Hz of selective refocusing bandwidth.

Selective-1D-spin-echo-TOCSY: The selective-1D-TOCSY experiments were performed with an added spin-echo block, in pseudo-2D mode, at five different spin-echo evolution times (0ms, 10ms, 30ms, 50ms, and 90ms). The data acquisition took around 2 min and 30s to complete.

Selective-1D-PSYCHE-TOCSY: The selective-1D-PSYCHE-TOCSY spectra were acquired in pseudo-2D mode with a recycle delay of 2s, 4 scans, and a low-flip angle of 15° for the double frequency swept pulses of 30ms length. The spectral widths of the direct and indirect dimensions were 8100 and 50 Hz, respectively. The direct and indirect dimensions respectively had 16k and 32 number of points. This experiment took around 6min 30s to acquire. The FID resolution for pure shift was 3.1 Hz. Isotropic mixing was produced by using a dipsi spin-lock block for 100ms.

Mixture of amino acids:
The required amino acid mixture sample was prepared by dissolving amino acids namely, leucine, isoleucine, valine, alanine, serine, threonine, aspartic acid, cysteine, proline, phenylalanine, and 4-OH-proline, 5-7mg of each in 500ul of D_2O. On this sample, 1D-^{1}H, spin-echo, PSYCHE, and TOCSY NMR spectra were acquired using an 800 MHz AVANCE-III NMR spectrometer equipped with a cryoprobe.

Experimental details:

1D-^{1}H: The proton 1D spectrum was acquired with a 2s recycle delay and 4 scans. The number of points was 32k, and the spectral width of 8100 Hz resulted in an FID duration of 2.02s (0.49 Hz of spectral resolution). The experiment took around 30s to acquire.

Spin-echo: The spin-echo 1D spectra were acquired with a 2s recycle delay, 4 scans, and five distinct spin-echo evolution times (0ms, 10ms, 30ms, 50ms, and 90ms). The number of points was 32k, and the spectral width of 8100 Hz resulted in an FID duration of 2.02s (0.49 Hz of spectral resolution). The experiment was carried out in pseudo-2D mode and took around 2min and 30s to acquire.

PSYCHE: The PSYCHE pure shift was acquired in pseudo-2D mode with a recycle delay of 2s, 4 scans, and a low-flip angle of 15° for the double frequency swept pulses of 30ms length. The spectral widths of the direct and indirect dimensions were 8100 and 50 Hz, respectively. The direct and indirect dimensions respectively had 16k and 32 number of points. This experiment took around 6 min and 30s to acquire. The spectral resolution for the pure shift was 3.1 Hz.

2D-TOCSY: Two-dimensional TOCSY experiments suitable for the FID-Net pure shift processing were acquired in pseudo-3D mode, with a recycle delay of 1s and 8 scans, and dipsi isotropic spinlock mixing set to 100 ms. The number of points in the direct and indirect

dimensions were 16k and 1024, respectively. At a spectral width of 8100 Hz along both dimensions, spectral resolution is 1 Hz along the direct and 16 Hz along the indirect dimensions. Prior to processing the data using FID-Net, Fourier transformation was performed along the indirect dimension, and all indirect slices were processed using the FID-Net model. The experiment took around 25 hours and 45 min to complete.

TSA 500 µM in DTT buffer solution:
The requisite sample was prepared in buffer containing 50 mM K_2HPO_4 pH 8.0, 30 mM KCl, 4 mM DTT, and 1 mM NaN_3 in 100% D_2O. This sample was utilized to record the necessary 1D-^{1}H, spin-echo, and PSYCHE NMR spectra using an 800 MHz AVANCE-III NMR spectrometer equipped with a cryoprobe.

Experimental details:

1D-^{1}H: The proton 1D spectrum was acquired with a 2s recycle delay and 64 scans. The number of points was 32k, with a spectral width of 8100 Hz resulting in an FID duration of 2.02 s (0.49 Hz spectral resolution). The experiment took around 4 min 30 sec to acquire.

Spin-echo: The spin-echo 1D spectra were acquired with a 2s recycle delay, 64 scans, and five distinct spin-echo evolution times (0ms, 10ms, 30ms, 50ms, and 90ms). The number of points was 32k, with a spectral width of 8100 Hz resulting in an FID duration of 2.02 s (0.49 Hz spectral resolution). The experiment was carried out in pseudo-2D mode and took around 27 min to acquire.

PSYCHE: The PSYCHE pure shift was acquired in pseudo-2D mode with a recycle delay of 2s, 64 scans, and a low-flip angle of 15° for the double frequency swept pulses of 30ms length. The spectral widths of the direct and indirect dimensions were 8100 Hz and 50 Hz, respectively. The direct and indirect dimensions respectively had 32k and 16 number of points. This experiment took around 1 hours 13 min to acquire. The resulting spectral resolution for the pure shift spectrum was 6.3 Hz.

6. Data processing:

All experimental NMR spectra were processed with NMRpipe[4] or using the Python libraries NMRGLUE[5] and NUMPY[6].

Supporting Tables

Table. S1: Parameters for the synthetic spin-echo NMR spectra simulation

**Parameter**	**Value**
Number of peaks	3 – 50 (uniform distribution)
Intensity	$N(1, 0.5)$[a]
Noise	$N(0.05, 0.025) \times$ (1 to 100)
^{1}H SW (Hz)	$N(10{,}000, 1{,}000)$
^{1}H number of complex points	$N(12{,}288, 16{,}384)$
$R_2(^1H)(s^{-1})$	$N(3, 2)$
Phase	$N(0, 1)$
J_{HH} (Hz)	$N(8, 3)$
$R_{2(inhom)}(^1H)(s^{-1})$	$N(1.5, 1)$

a) $N(\mu, \sigma)$ is a normal distribution with mean μ and standard deviation σ

Supporting Figures

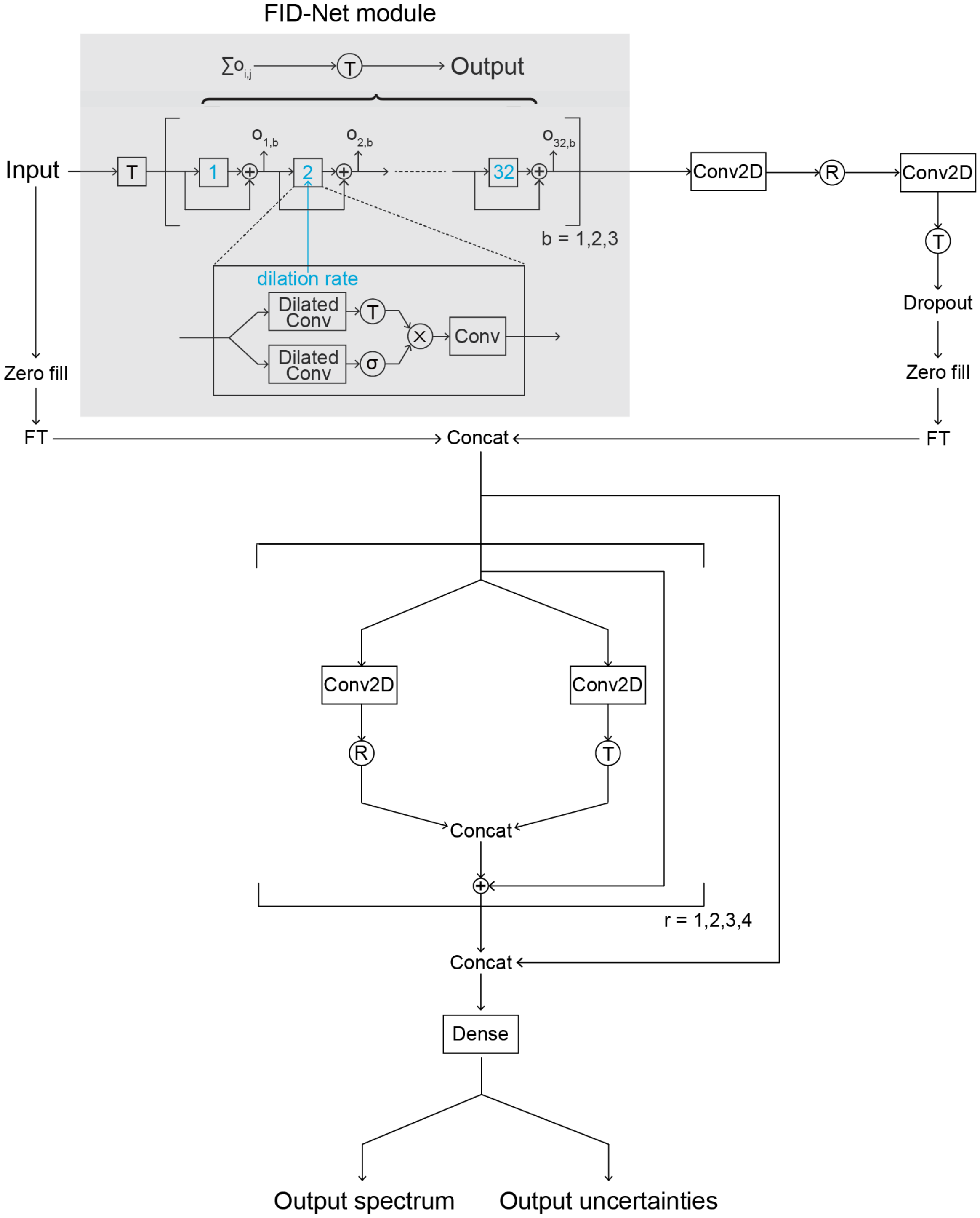

Figure S1. The complete FID-Net-PS DNN architecture for obtaining pure shift ^{1}H NMR spectra in a broadband manner. The FID-Net block (highlighted in grey) is very similar to the FID-Net model published earlier.[3] Circles represent the elementwise transformations; T: *tanh*() operator, R: rectified linear unit, σ: sigmoidal operator, +: summation, ×: multiplication. During these operations weights are not updated, whereas rectangles represent layers with trainable weights; Conv: Convolution layer, Conv2D: two-dimensional convolution layer; Dense: dense layer, Dropout: 10% dropout is applied during the training. Fourier Transformation is represented with FT.

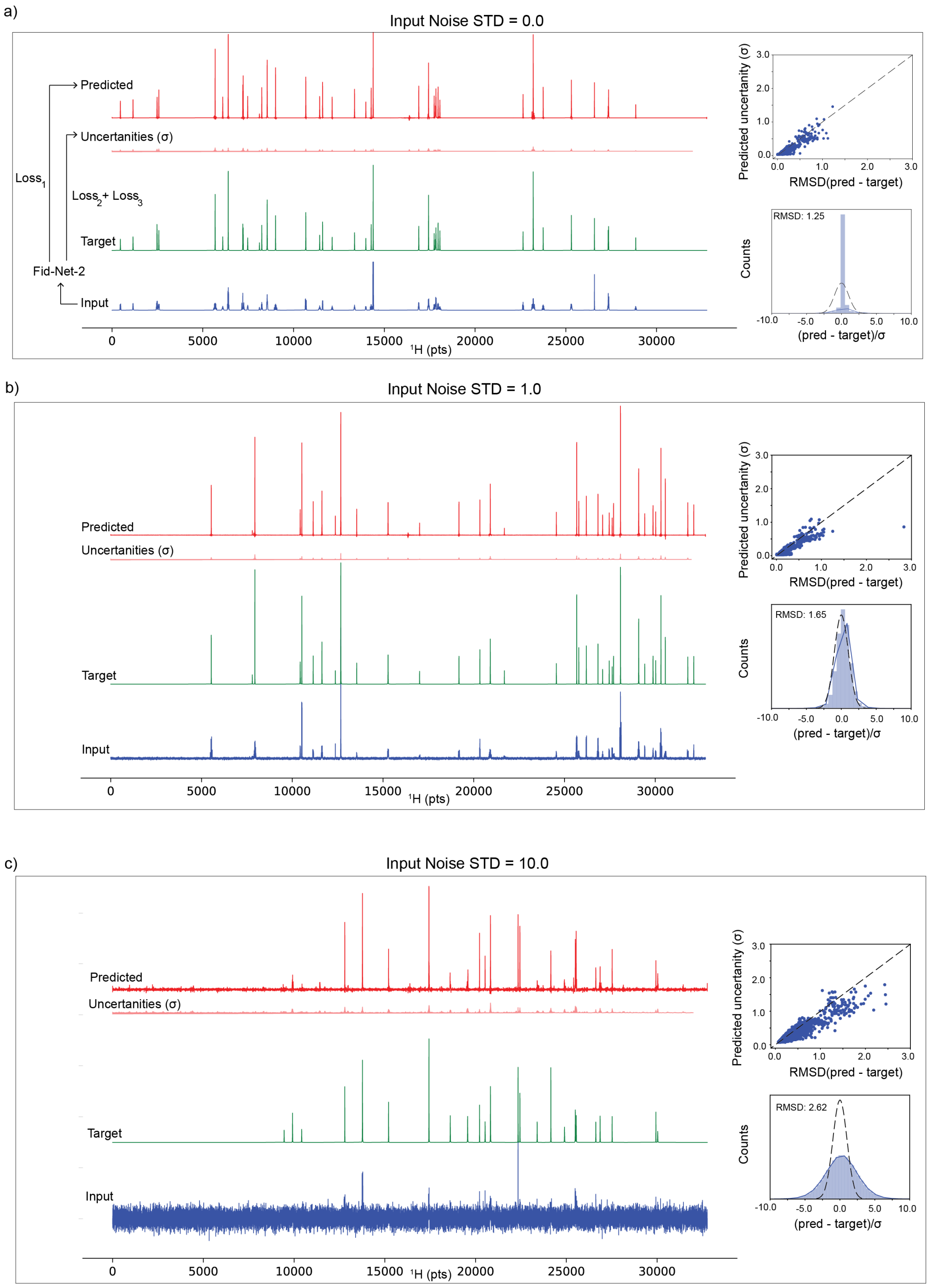

Figure S2: Evaluation of the pure shift FID-Net-PS model using synthetic NMR datasets. A total of 100 randomly generated NMR spectra at each noise level were used for this assessment. (a) Spin-echo spectra with a noise standard deviation of 0.0 were used as inputs to the pure shift FID-Net-PS model, which then predicted the corresponding pure shift spectra along with their point-by-point uncertainties. The predicted pure shift spectrum closely matches the target pure shift spectrum, demonstrating the model's high fidelity. The correlation between RMSD(*predict* – *target*) and the predicted uncertainties, as well as the deviation from the standard normal distribution of residuals, reveals that the uncertainties are slightly underestimated, which is an expected outcome since the model was trained over a broad range of noise levels. Panel (b) and (c) show similar behaviour for noise standard deviations of 1.0 and 10.0, respectively. The evaluation across different noise levels highlights the systematic trend of underestimation, which provides a basis for calibrating the model's uncertainty estimations. For plotting, the $(\text{predict} - \text{target})/\sigma$ values are clipped to the range $[-10{,}10]$ to remove outliers.

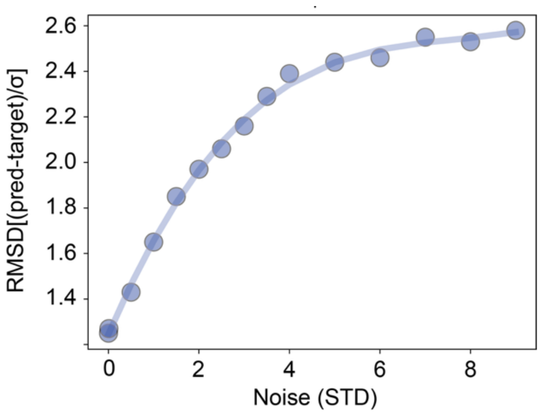

Figure S3: Calibration of uncertainty estimation using RMSD((*predict* – *target*)/σ). These calculations were performed on 100 synthetic spectra with noise added with standard deviations ranging from 0.0 to 10.0 in the input spin-echo NMR spectra. The resulting RMSD((*predict* – *target*)/σ) values serve as correction factors for σ at the corresponding noise levels of the input spectra. The data were fitted to a third-order polynomial and the resulting coefficients are subsequently used to derive σ correction factors for any given noise level. All experimental NMR datasets were subsequently processed using these calibrated σ correction factors to improve the reliability of the uncertainty estimates.

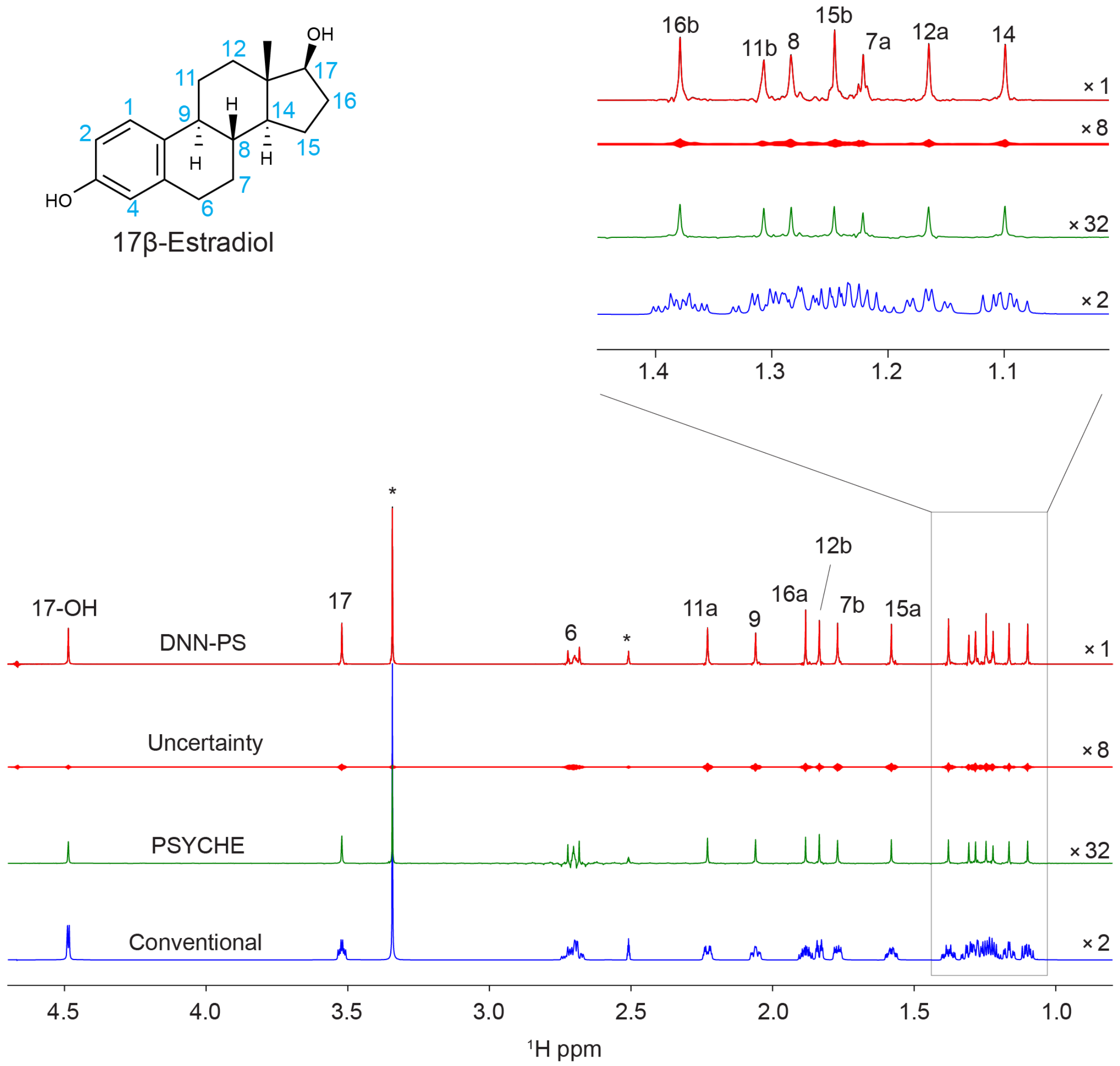

Figure S4: Comparison of conventional, deep neural network yielded pure shift (DNN-PS), uncertainties obtained from the DNN, and PSYCHE homonuclear decoupled 1D ^{1}H-NMR spectra recorded on 73.4 mM 17β-estradiol (in DMSO-D_6). The * symbols represent the solvent residual signals.

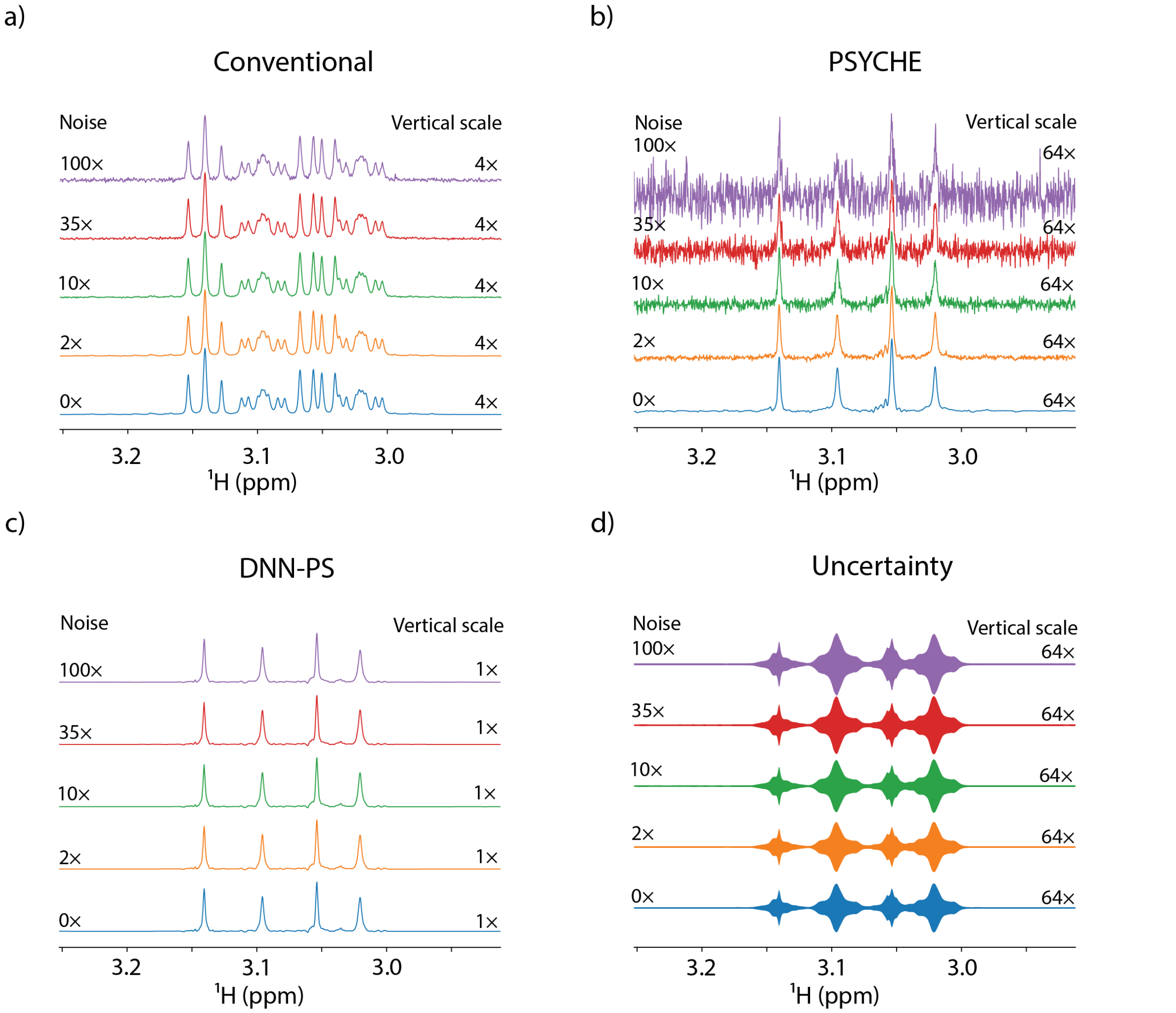

Figure S5: Evaluation of the signal-to-noise ratio (S/N) in pure shift NMR spectra of Kanamycin. (a) Conventional ^{1}H 1D, (b) PSYCHE, (c) FID-Net-PS (DNN-PS), and (d) predicted uncertainty spectra. The relative added noise factors (with respect to the original noise) to the PSYCHE and input spin-echo spectra for DNN-PS are indicated. It is evident that the spectral quality of PSYCHE degrades substantially with increasing noise, whereas the DNN-PS output spectra remain remarkably stable, even when the input spin-echo spectra contain up to 100-fold noise. This stems from the fact that the majority of the uncertainty of the DNN-PS spectrum originates from model uncertainty.

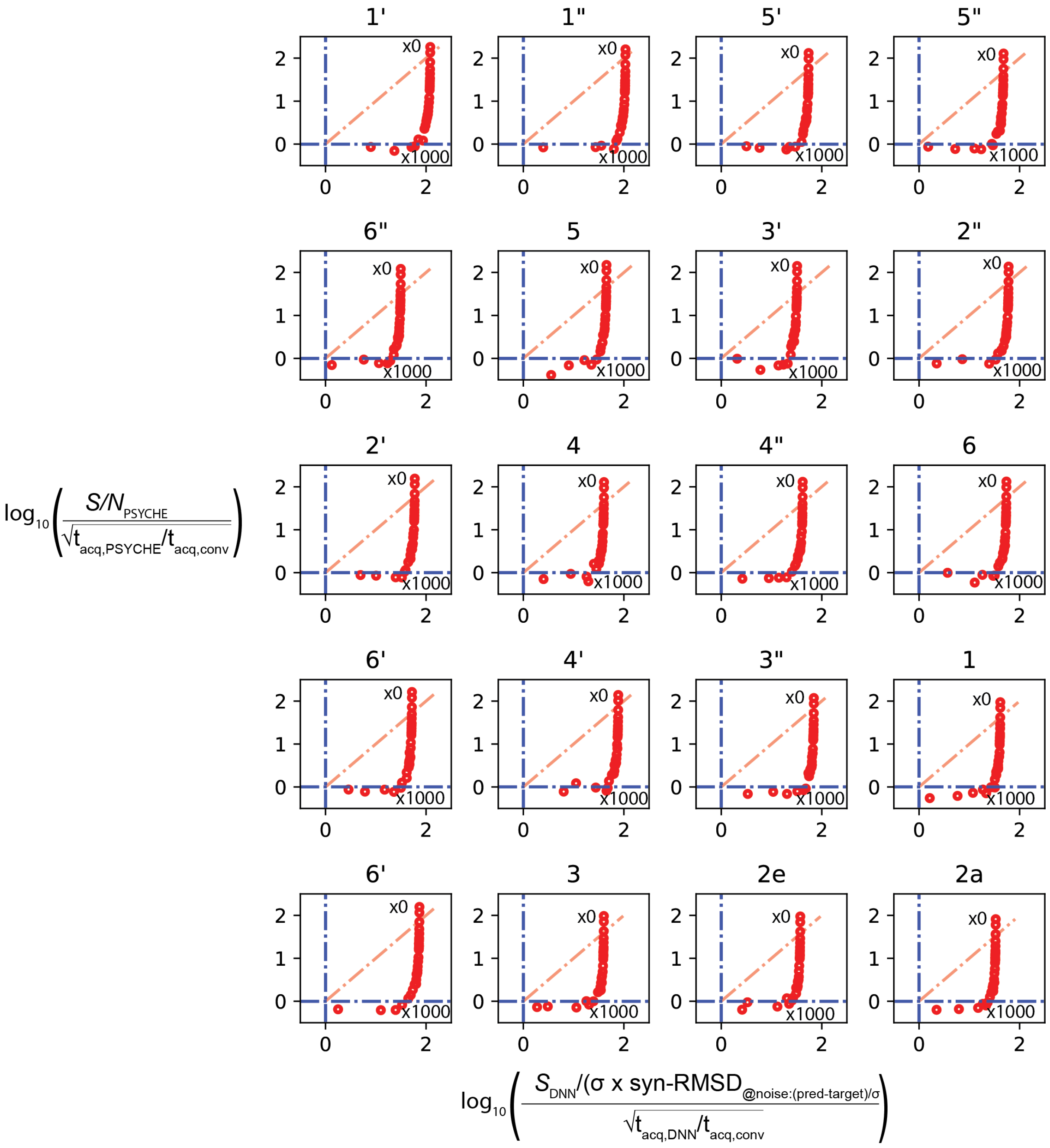

Figure S6. Comparison of normalized signal-to-noise ratios (S/N) between PSYCHE and DNN-PS spectra. The normalized S/N of PSYCHE is compared with that of DNN-PS, where the latter is calculated as the peak intensity to uncertainty ratio (σ, calibrated at the corresponding noise levels). Different levels of added noise (multiples of the original spectral noise) are indicated in each subplot. The square roots of the experimental time ratios between PSYCHE and conventional spectra, and between spin-echo and conventional spectra, were used as normalization factors for PSYCHE and DNN-PS, respectively. The results show that the spectral sensitivity of DNN-PS remains stable across all peaks even with additional noise added, whereas the S/N of PSYCHE spectra decreases substantially with increasing noise.

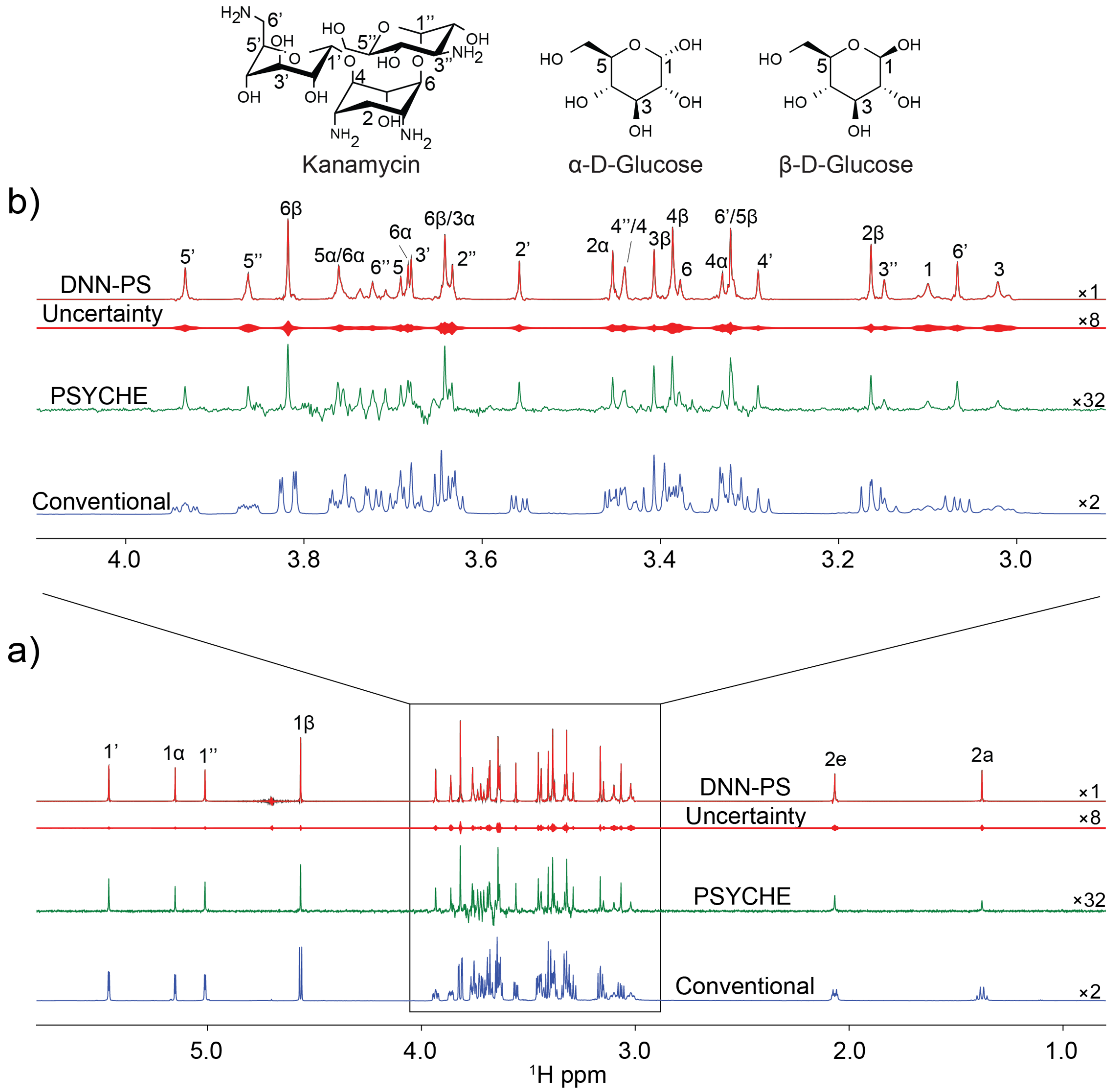

Figure S7: Comparison of conventional, deep neural network yielded pure shift (DNN-PS) and uncertainties obtained from the DNN, as well as PSYCHE homonuclear-decoupled 1D ^{1}H-NMR spectra recorded on (a) a mixture of kanamycin and glucose (3.4 mM + 11.1 mM, dissolved in D_2O). (b) The expansion of an overlapped chemical shift region. This comparison demonstrates that the DNN-derived pure shift NMR spectral resolution is substantially better than the conventional 1D ^{1}H NMR spectrum, and the spectral sensitivity is better than the PSYCHE pure shift spectrum. The predicted uncertainties are, as expected, relatively larger for the severely overlapped chemical shift regions.

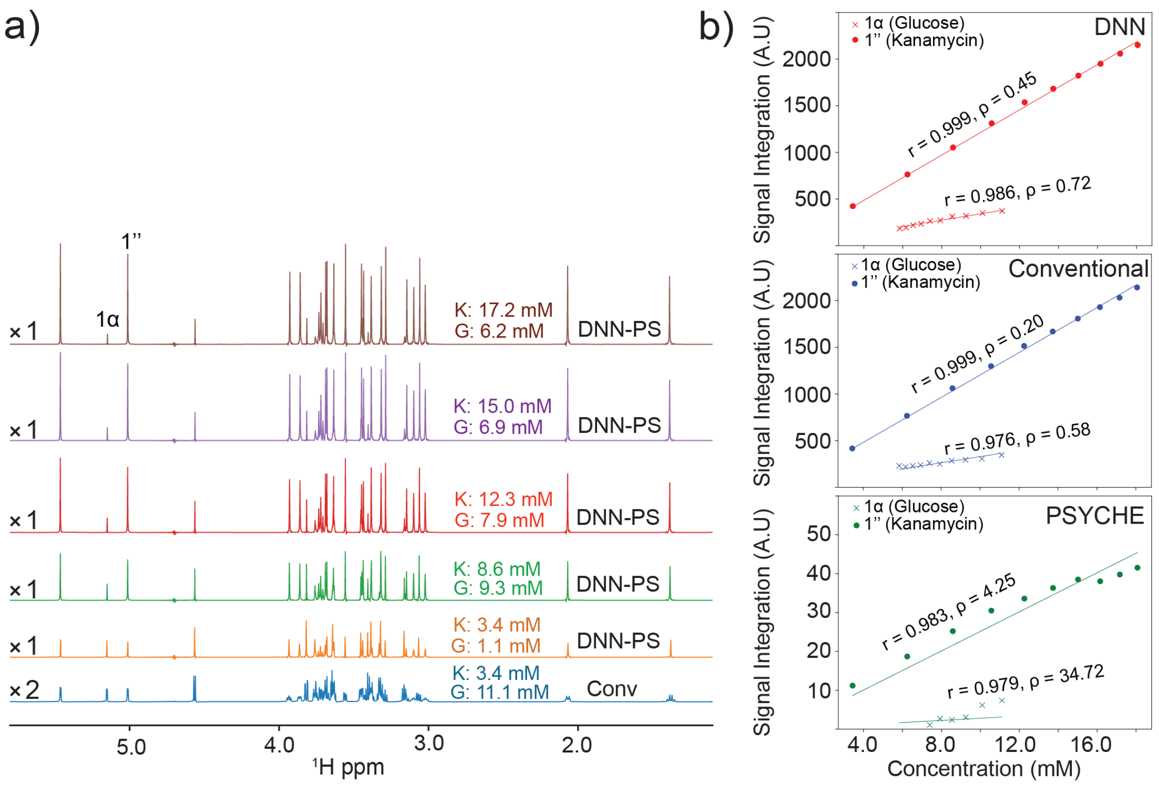

Figure S8: (a) The conventional and DNN-PS 1D-^{1}H NMR spectra of kanamycin (K) and glucose (G) mixtures at different concentration ratios. The resonances marked with the cross and filled circle, respectively, are used to calculate the concentrations of glucose and kanamycin. (b) The concentrations were derived from these resonances using various approaches: conventional (blue), DNN-PS (red), and PSYCHE (green), were integrated and presented against sample concentrations (kanamycin (filled circles) and glucose (cross)). Pearson correlation coefficients (r) for the DNN-PS and PSYCHE range between 0.980 and 0.999, which is satisfactory for quantification, and the second metric, normalized RMSD, $\tilde{\rho}$ is rather smaller for the DNN-PS resonances when compared with PSYCHE signals.

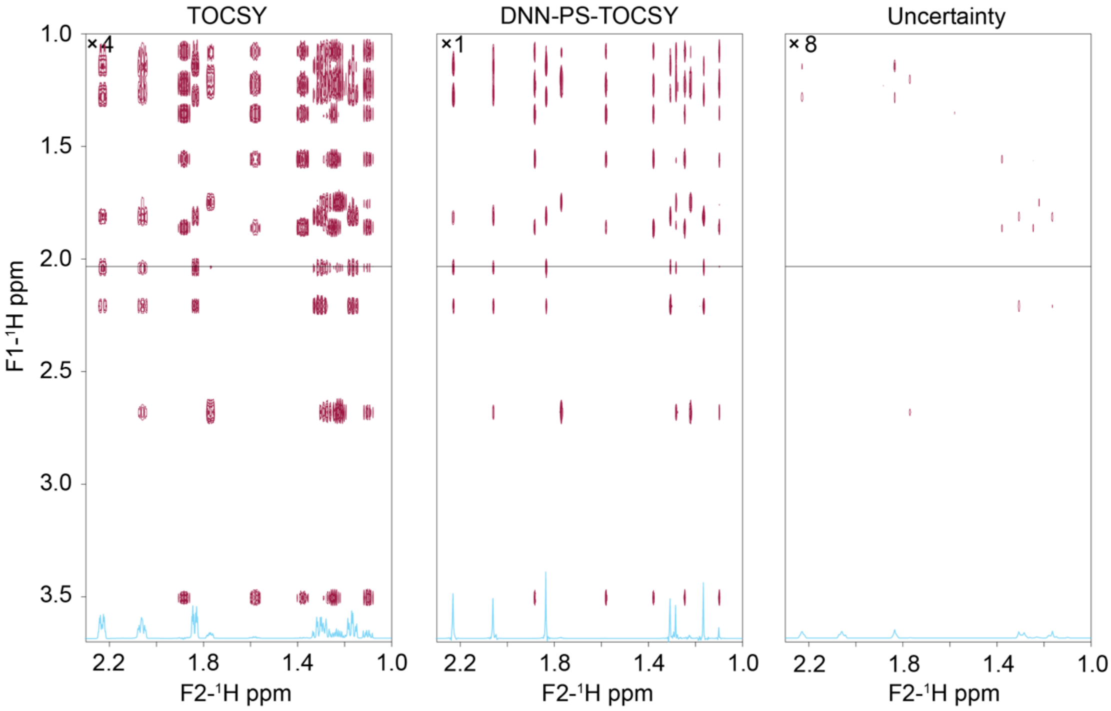

Figure S9: Comparison of two-dimensional TOCSY spectra of estradiol in conventional ^{1}H and DNN-PS modes. The conventional 2D-TOCSY has a significant peak overlapping; however, all these overlaps are eliminated in the DNN-PS processed TOCSY spectra, revealing obvious TOCSY correlations. Internal projections also show improved spectral resolution and sensitivity for the DNN-PS TOCSY compared with conventional TOCSY. The threshold value for the conventional TOCSY is set 4 times lower than the DNN-PS, and the predicted uncertainties are also plotted.

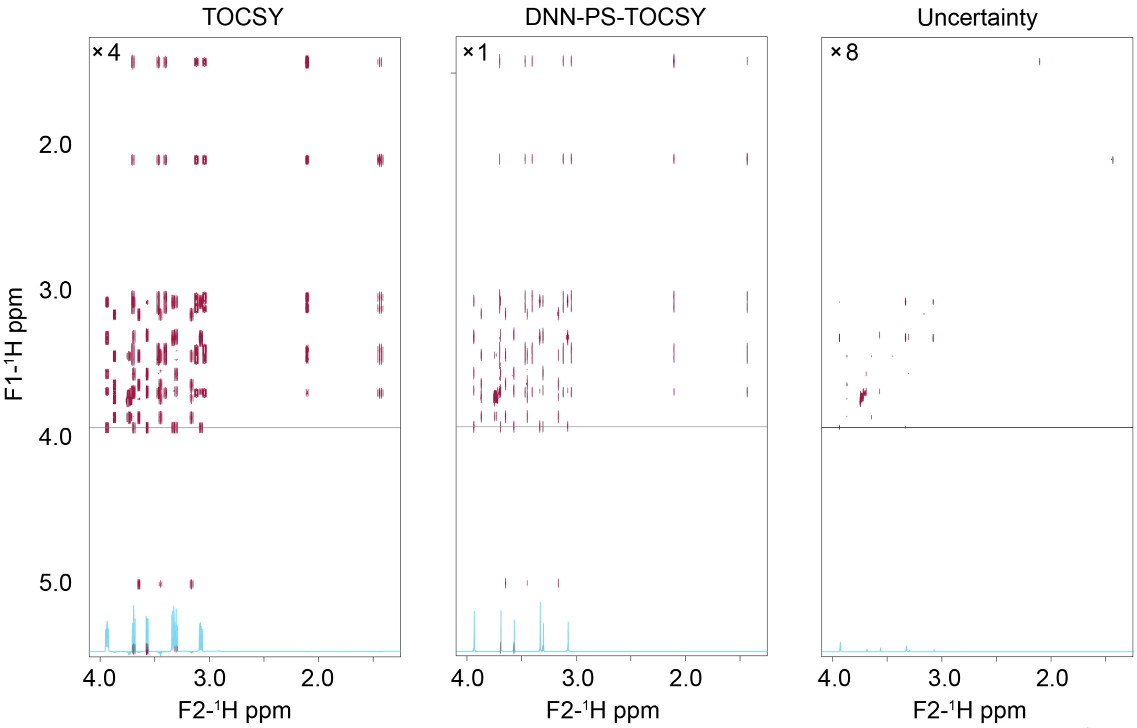

Figure S10: Comparison of two-dimensional TOCSY spectra of kanamycin in conventional ^{1}H and DNN-PS modes. The conventional 2D-TOCSY has a significant overlap of peaks; however, all these overlaps are eliminated in the DNN-PS processed TOCSY spectra, revealing obvious TOCSY correlations. Internal projections also show improved spectral resolution and sensitivity for the DNN-PS TOCSY compared with conventional TOCSY. The threshold value for the conventional TOCSY is set 4 times lower than the DNN-PS, and the predicted uncertainties are also plotted.

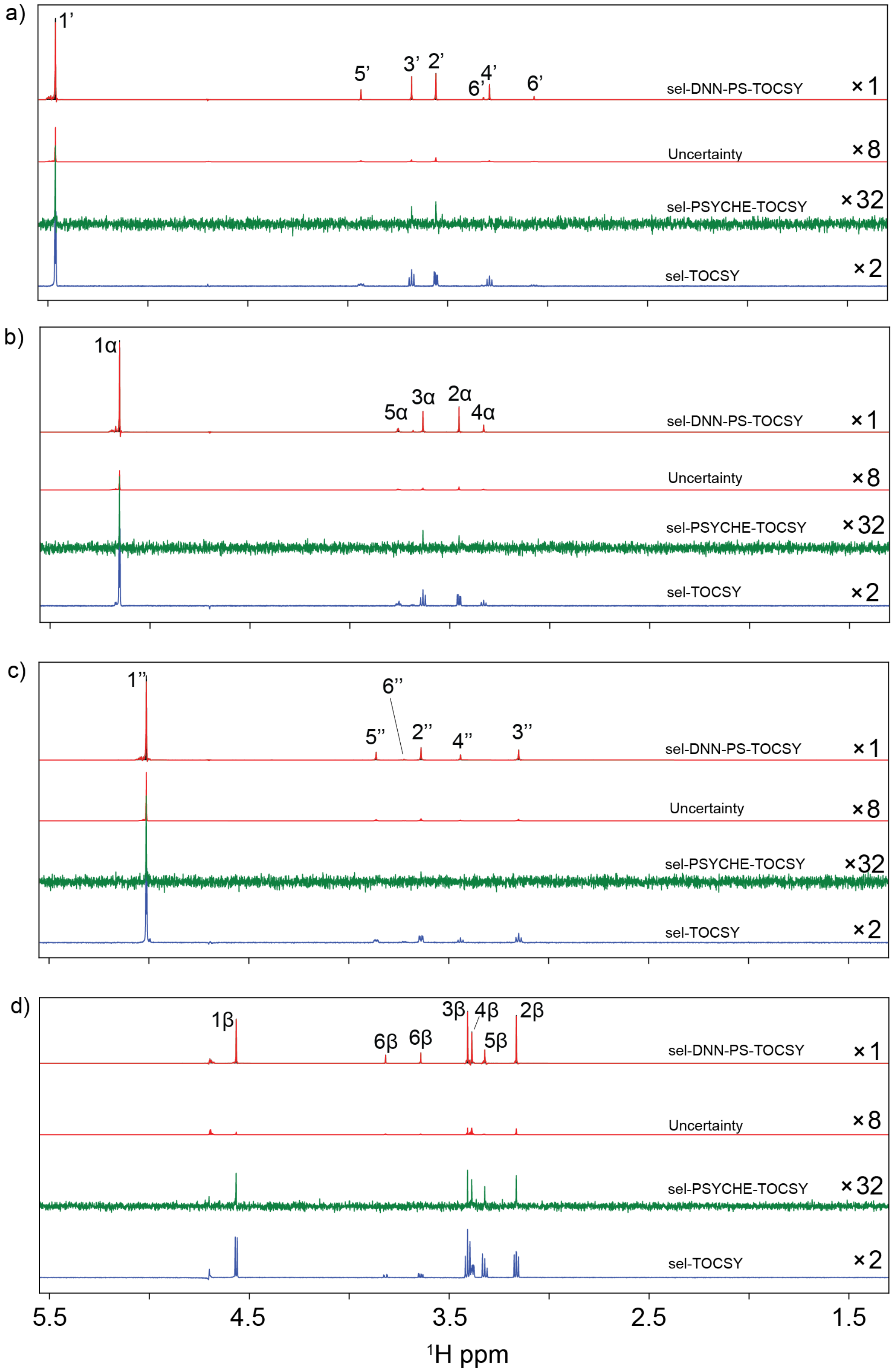

Figure S11: When applicable, 1D TOCSY experiments are substantially faster to monitor chemical shift correlations in mixtures compared to 2D TOCSY experiments. In this regard, selective 1D-TOCSY experiments were performed using conventional, PSYCHE, and DNN-PS detection, on the mixture of kanamycin (3.4 mM) and glucose (11.1 mM). The conventional selective-TOCSY has a significant resonance overlap; however, all these complex resonance multiplet patterns are simplified to singlets in the DNN-PS processed selective-TOCSY spectra, revealing obvious TOCSY correlations. On the other hand, selective PSYCHE-TOCSY spectra suffer from very poor spectral sensitivity, which hinders the detection of all the TOCSY correlations.

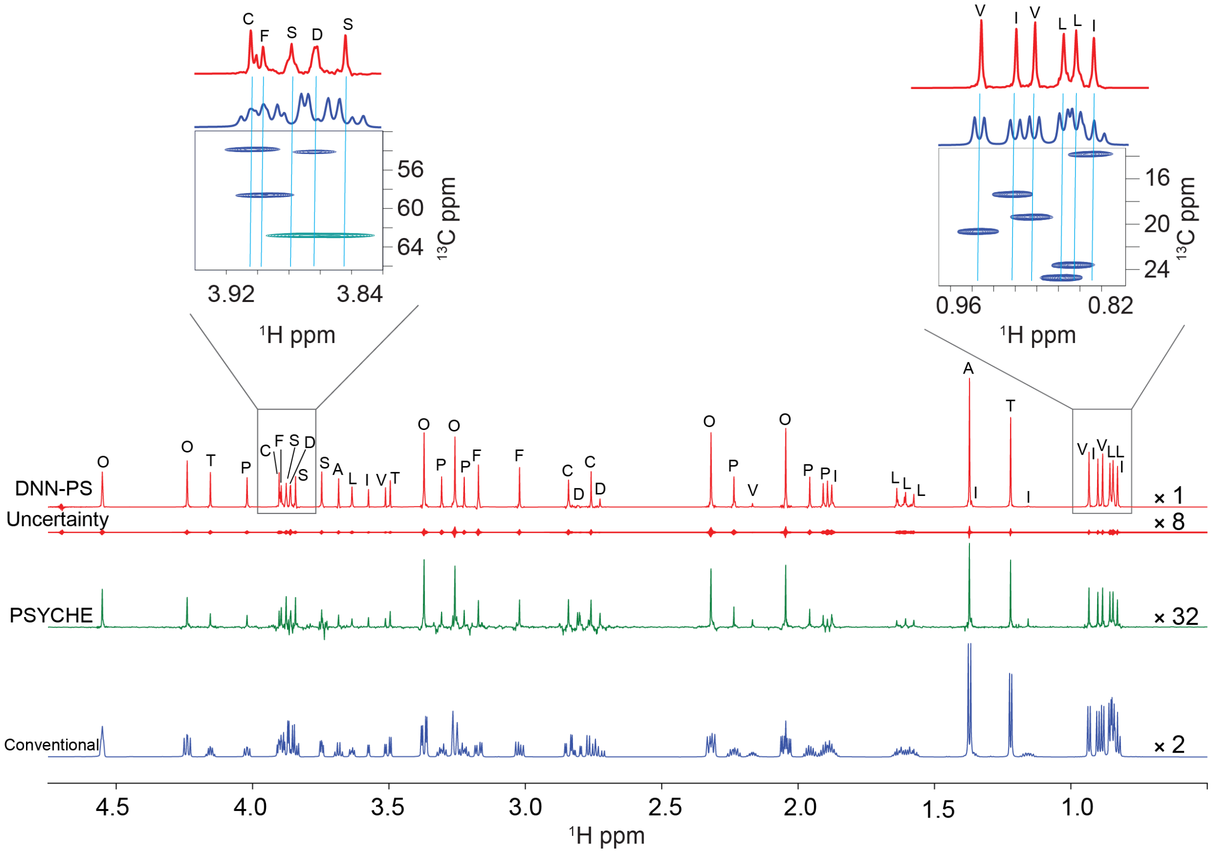

Figure S12: Comparison of conventional, deep neural network yielded pure shift (DNN-PS), uncertainties obtained from the FID-Net, and PSYCHE homonuclear-decoupled 1D ^{1}H-NMR spectra recorded on a mixture of 11 different amino acids (leucine, isoleucine, valine, alanine, serine, threonine, aspartic acid, cysteine, proline, phenylalanine, and 4-OH-proline; 5-7 mg of each in 500 µl D_2O) on an 800 MHz NMR spectrometer. The spectral sensitivity of the DNN-PS spectrum is clearly higher than that of the PSYCHE, and it does not severely suffer from the strong-coupling artifacts as in the PSYCHE. Peak positions from the expanded DNN-PS spectra are compared to HSQC contour positions (vertical lines) for two of the crowded chemical shift regions, and they are found to be well correlated, indicating that the chemical shifts generated by the DNN-PS are correct.

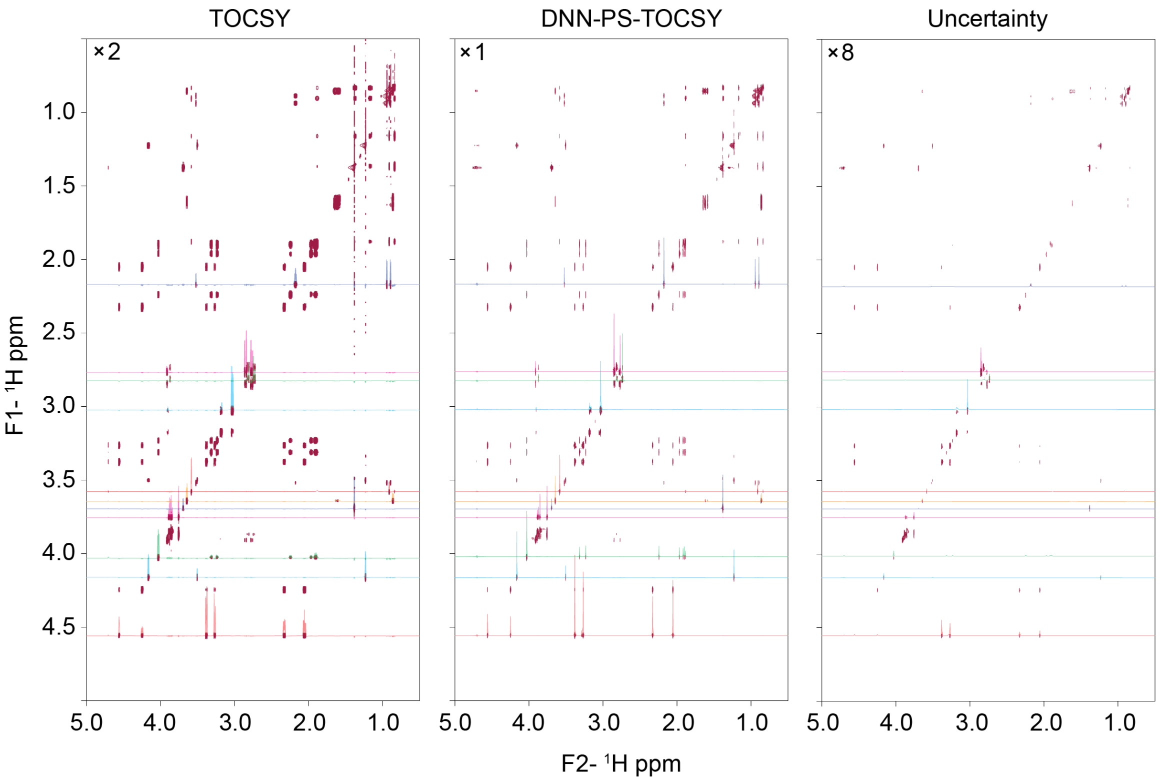

Figure S13: Comparison of two-dimensional TOCSY recorded in conventional, deep neural network yielded pure shift (DNN-PS), and uncertainties obtained from the FID-Net-PS on a mixture of 11 different amino acids (leucine, isoleucine, valine, alanine, serine, threonine, aspartic acid, cysteine, proline, phenylalanine, and 4-OH-proline; 5-7 mg of each in 500 µl D_2O) on an 800 MHz NMR spectrometer. All TOCSY correlations can be clearly seen in the projections when the data is processed with FID-Net-PS, which is not feasible in conventional experiments.

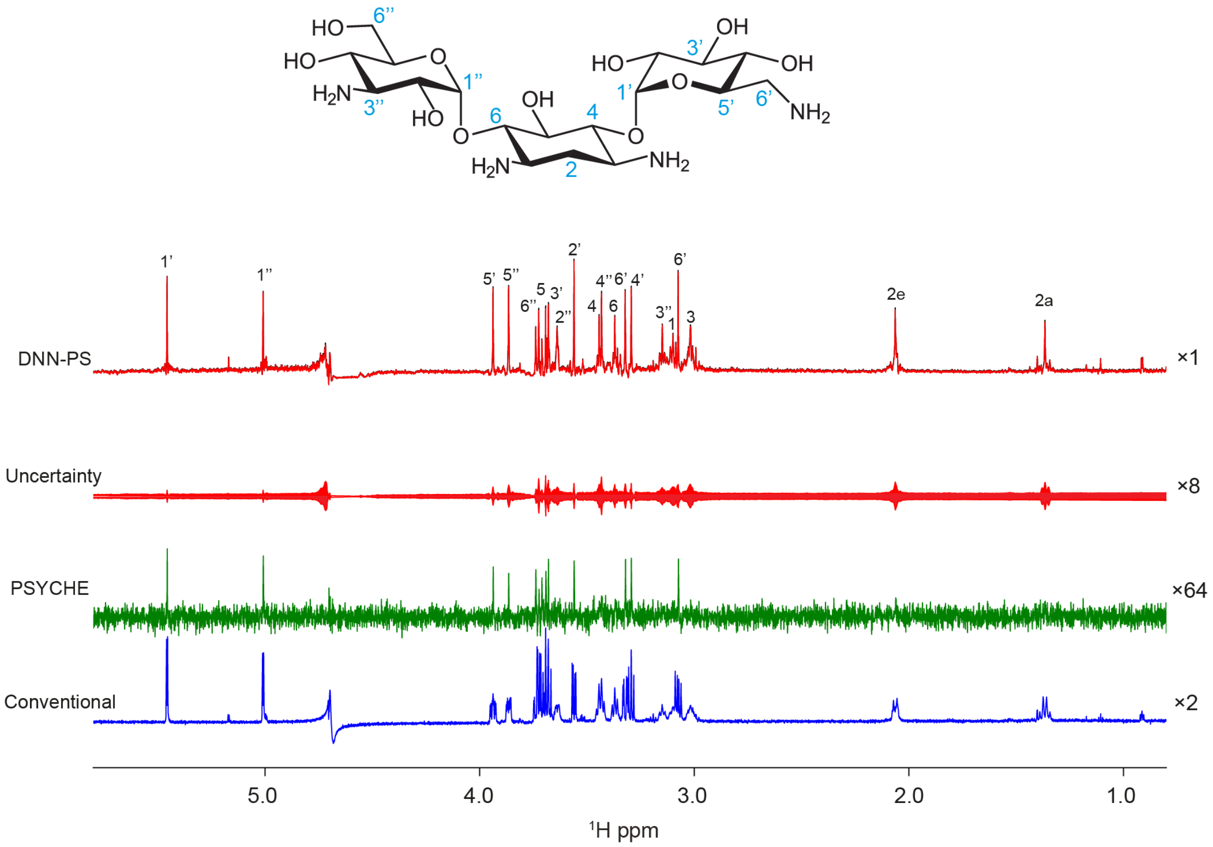

Figure S14: Comparison of conventional, deep neural network yielded pure shift (DNN-PS), uncertainties obtained from the DNN, and PSYCHE homonuclear-decoupled 1D ^{1}H-NMR spectra recorded on 50 µM kanamycin (in D_2O). Several peaks in the conventional spectrum appear broadened due to inherent conformational exchange at the lower concentration of kanamycin, which hinders their detection in the PSYCHE spectrum. On the other hand, due to the higher sensitivity of FID-Net-PS, all such broad signals were still detectable. Nevertheless, improper spin-echo modulation caused by dominant line broadening resulted in residual splitting patterns. The predicted uncertainties in this case are relatively larger than those obtained for the 34.7 mM kanamycin sample, reflecting the higher noise levels associated with lower sample concentrations. Similarly, the broadened resonances exhibit relatively larger predicted uncertainties than the sharper lines.

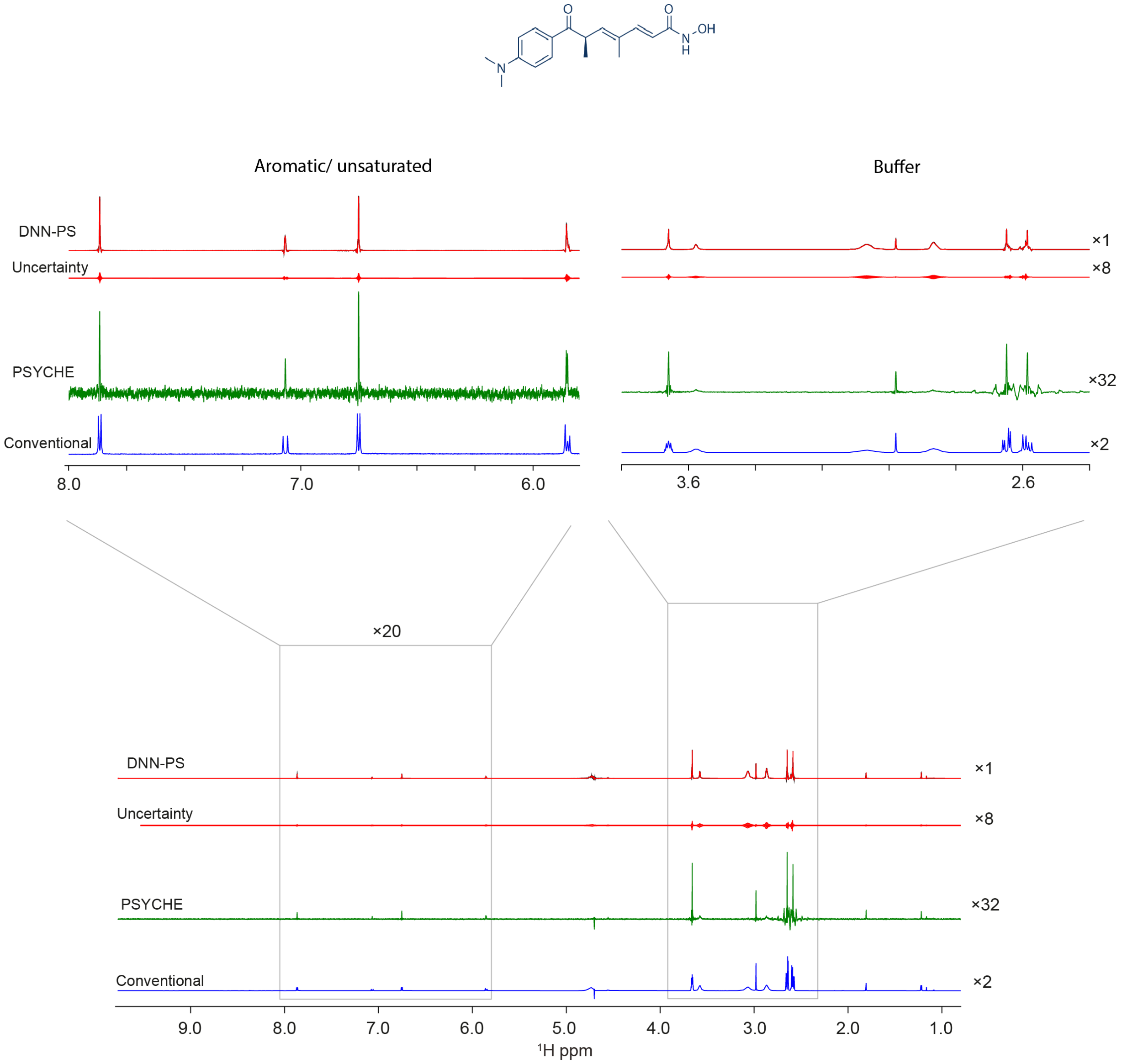

Figure. S15: Comparison of conventional, deep neural network yielded pure shift (DNN-PS), uncertainties obtained from the DNN, and PSYCHE homonuclear-decoupled 1D ^{1}H-NMR spectra recorded on 500 μM trichostatin A (DTT buffer solution in D_2O solvent). A few peaks in the conventional spectrum appear broadened due to inherent –OH and –SH chemical exchange, which hampers their detection in the PSYCHE spectrum (30ms duration of the double frequency-swept pulses). In contrast, all these broad signals were preserved in the DNN-PS spectrum.

Supporting References

(1) Martín, A.; Ashish, A.; Paul, B.; Eugene, B.; Zhifeng, C.; Craig, C.; Greg, S. C.; Andy, D.; Jeffrey, D.; Matthieu, D.; Sanjay, G.; Ian, G.; Andrew, H.; Geoffrey, I.; Michael, I.; Jia, Y.; Rafal, J.; Lukasz, K.; Manjunath, K.; Josh, L.; Dandelion, M.; Rajat, M.; Sherry, M.; Derek, M.; Chris, O.; Mike, S.; Jonathon, S.; Benoit, S.; Ilya, S.; Kunal, T.; Paul, T.; Vincent, V.; Vijay, V.; Fernanda, V.; Oriol, V.; Pete, W.; Martin, W.; Martin, W.; Yuan, Y.; Xiaoqiang, Z. TensorFlow: Large-Scale Machine Learning on Heterogeneous Systems. 2015. https://www.tensorflow.org/.

(2) Shukla, V. K.; Karunanithy, G.; Vallurupalli, P.; Hansen, D. F. Characterising Aromatic Side Chains in Proteins through the Synergistic Development of NMR Experiments and Deep Neural Networks. bioRxiv 2024, 2024.04.01.587635. https://doi.org/10.1101/2024.04.01.587635.

(3) Karunanithy, G.; Hansen, D. F. FID-Net: A Versatile Deep Neural Network Architecture for NMR Spectral Reconstruction and Virtual Decoupling. J Biomol NMR 2021, 75 (4–5), 179–191. https://doi.org/10.1007/s10858-021-00366-w.

(4) Delaglio, F.; Grzesiek, S.; Vuister, GeertenW.; Zhu, G.; Pfeifer, J.; Bax, A. NMRPipe: A Multidimensional Spectral Processing System Based on UNIX Pipes. J Biomol NMR 1995, 6 (3). https://doi.org/10.1007/BF00197809.

(5) Helmus, J. J.; Jaroniec, C. P. Nmrglue: An Open Source Python Package for the Analysis of Multidimensional NMR Data. J Biomol NMR 2013, 55 (4), 355–367. https://doi.org/10.1007/s10858-013-9718-x.

(6) Harris, C. R.; Millman, K. J.; van der Walt, S. J.; Gommers, R.; Virtanen, P.; Cournapeau, D.; Wieser, E.; Taylor, J.; Berg, S.; Smith, N. J.; Kern, R.; Picus, M.; Hoyer, S.; van Kerkwijk, M. H.; Brett, M.; Haldane, A.; del Río, J. F.; Wiebe, M.; Peterson, P.; Gérard-Marchant, P.; Sheppard, K.; Reddy, T.; Weckesser, W.; Abbasi, H.; Gohlke, C.; Oliphant, T. E. Array Programming with NumPy. Nature 2020, 585 (7825), 357–362. https://doi.org/10.1038/s41586-020-2649-2.
